# Silver Nanoparticles from *Stryphnodendron adstringens*: A Green Synthesis Approach, Characterization, and Their Impact on Bacterial Metabolism

**DOI:** 10.1021/acsomega.6c06132

**Published:** 2026-08-14

**Authors:** Maelson Cardoso Lacerda, Thyerre Santana da Costa, Uedson da Silva das Neves, Symone Costa de Castro, Andrei Alaferdov, Raluca Savu, Ljubica Tasic

**Affiliations:** † Laboratory of Biological Chemistry (LQB), Institute of Chemistry, 28132 Universidade Estadual de Campinas (UNICAMP), Campinas, São Paulo 13083-970, Brasil; ‡ National Institute of Science and Technology of Bioanalytics − Lauro Kubota (INCT-Bio L.K.), Universidade Estadual de Campinas (UNICAMP), Campinas, São Paulo 13083-970, Brasil; § Center for Semiconductor Components and Nanotechnologies (CCS Nano), Universidade Estadual de Campinas (UNICAMP), Campinas, São Paulo 13083-870, Brazil

## Abstract

This study reports the green synthesis of silver nanoparticles (AgNP@SA) using an extract from the medicinal plant *Stryphnodendron adstringens* as both the reducing and stabilizing agent. The resulting nanoparticles were spherical, with an average diameter of 12 ± 2 nm, and exhibited high colloidal stability, as indicated by a zeta potential of −28 ± 6 mV. Their formation and physicochemical properties were confirmed by UV–vis, TEM, EDS, STEM, DLS, XPS, FT-IR, UHPLC-HRMS/MS, and ^1^H-NMR analyses. Spectroscopic data showed that tannins and other phenolic compounds in the plant extract were primarily responsible for the reduction of Ag^+^ ions and stabilization of the nanoparticles, pointing to the involvement of related flavonoid derivatives. UHPLC-HRMS/MS analysis, interpreted through the GNPS database, further demonstrated the absence of epigallocatechin in the final nanoparticle system, supporting its proposed role as the reducing agent during nanoparticle synthesis. Biological assays demonstrated that AgNP@SA exhibited broad-spectrum antimicrobial activity against both Gram-positive and Gram-negative bacteria. ^1^H-NMR-based metabolomic profiling of *Staphylococcus aureus* under AgNP@SA stress revealed significant metabolic disruption, including decreased levels of leucine and valine and increased levels of lysine, acetate, and betaine, suggesting the induction of cellular stress and impairment of essential metabolic pathways. Overall, these findings highlight the potential of green-synthesized AgNP@SA as effective antimicrobial agents and reinforce the value of plant-mediated nanoparticle synthesis for biomedical applications.

## Graphical abstract

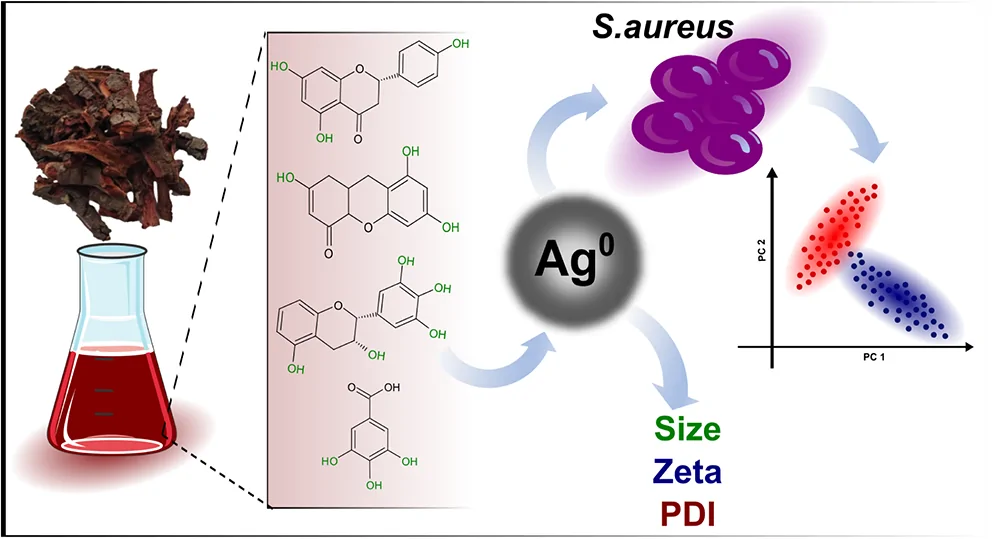

## 1 Introduction

In recent years, nanotechnology has emerged as a promising field of research with the potential to revolutionize many areas, from medicine to electronics. Among the synthesized nanomaterials, silver nanoparticles (AgNPs) have attracted considerable attention due to their unique properties, such as antimicrobial, antiviral, and antifungal activities, making them ideal candidates for biomedical applications.^1–3^

AgNPs can be synthesized using a variety of methods, including chemical, physical, and biological routes. However, conventional chemical and physical methods for silver nanoparticle synthesis often rely on hazardous reducing agents, such as sodium borohydride, hydrazine, and dimethylformamide, as well as organic solvents and synthetic stabilizers. In addition, these methods frequently require energy-intensive conditions, including high temperatures, elevated pressures, prolonged reaction times, or irradiation (e.g., UV, microwave, or laser), resulting in nanoparticles with reduced biocompatibility and increased environmental impact.^4^ In this context, the green synthesis of AgNPs, which uses plant extracts as reducing agents, has emerged as a promising alternative, as it is an environmentally friendly, low-cost method that produces nanoparticles with high biocompatibility and stability.^5,6^

In this study, the extract of *Stryphnodendron adstringens* (barbatimão) was selected as a reducing and stabilizing agent for the green synthesis of silver nanoparticles, owing to its well-documented antimicrobial activity and high content of bioactive polyphenols.^7–9^ The bark is particularly rich in hydrolyzable and condensed tannins, flavonoids (i.e., epigallocatechin, quercetin and rutin), alkaloids, triterpenes, steroids, and organic acids, components that contribute to its biological properties.^10,11^ Tannins and flavonoids are recognized for their antioxidant, anti-inflammatory, and antimicrobial activities, acting through protein precipitation, disruption of microbial cell membranes, and interference with essential cellular processes.^12–15^ Studies report that the extract possesses a broad spectrum of antimicrobial activity against Gram-positive bacteria, including *Staphylococcus aureus* and *Staphylococcus epidermidis*; Gram-negative bacteria, such as *Escherichia coli* and *Pseudomonas aeruginosa*; and the fungus *Candida albicans*.^14,15^ These characteristics make *S. adstringens* an attractive phytochemical source for the green synthesis of functionalized silver nanoparticles (AgNPs) with enhanced antimicrobial potential.

Recent literature has shown growing interest in the green synthesis of AgNPs using extracts from medicinal plants. Several studies have investigated the potential of different plants, such as *Azadirachta indica*,^16^ *Aloe vera*,^17^ *Camellia sinensis*,^18^ and sunflower extracts (*Helianthus annuus*),^19^ for the synthesis of AgNPs with antimicrobial properties and other biomedical applications. However, the application of *S. adstringens* for the synthesis of AgNPs remains largely underexplored; the novelty of this work lies in integrating green nanotechnology with mechanistic biological evaluation. Specifically, this work demonstrates the feasibility of synthesizing stable AgNPs using a tannin- and phenolic-rich *S. adstringens* extract and systematically correlates their physicochemical properties with antimicrobial efficacy and metabolomic alterations in bacterial cells, providing new insights into the mechanisms underlying their antibacterial activity. By integrating physicochemical characterization with biological and metabolomic analyses, this work provides new insights into the potential of sustainably synthesized AgNPs for biomedical applications, highlighting an environmentally friendly approach to nanomaterial development.

## 2 Materials and Methods

### 2.1 Chemicals and Reagents

Bark samples from mature *S. adstringens* trees were collected in an area between the municipalities of Mamonas, Monte Azul, Gameleiras, and Espinosa in the state of Minas Gerais, Brazil. This area is located between the coordinates 14°48′S, 43°06′W and 15°18′S, 42°50′W, and was subsequently registered in the *Sistema Nacional de Gestão do Patrimônio Genético e do Conhecimento Tradicional Associado* SisGen (National System for the Management of Genetic Heritage and Associated Traditional Knowledge) under the code A54AFFC. All reagents were of analytical or chromatographic grade and used as received without further purification. Whatman No. 1 filter paper was obtained from Cytiva. Silver nitrate (AgNO_3_, ≥ 99.9%, trace metals basis) and sodium hydroxide (NaOH, ≥ 98%, ACS reagent grade) were purchased from Sigma-Aldrich. Deuterium oxide (D_2_O, 99.9 atom % D) was supplied by Cambridge Isotope Laboratories. Formic acid (≥ 98%, ACS reagent) and acetonitrile (≥ 99.9%, HPLC grade) were obtained from Merck. Methanol (≥ 99.9%, HPLC grade) was purchased from Sigma-Aldrich. Mueller–Hinton agar, Luria–Bertani (LB) medium, and Brain Heart Infusion (BHI) agar (microbiological grade) were supplied by BD Difco. Phosphate-buffered saline (PBS, pH 7.4, sterile, analytical grade) was obtained from Sigma-Aldrich. All reagents were acquired through authorized distributors in Brazil, ensuring high purity, batch traceability, and reproducibility in accordance with international analytical and microbiological standards. The bacterial strains were sourced from the Tropical Culture Collection of the André Tosello Foundation (Campinas, São Paulo, Brazil) and used for antimicrobial activity assessment.

### 2.2 Plant Extract

The material was washed with distilled water, dried at 25 ± 2 °C for 7 days, and ground in a knife mill with stainless steel blades. Two grams of the resulting powder were added to 50 mL of ultrapure water (Milli-Q system), transferred to amber bottles, and kept in maturation for 72 h, protected from light. The extract was filtered through No. 1 filter paper and stored at 4 °C until use.

### 2.3 Synthesis of AgNP@SA

The synthesis of the silver nanoparticles was performed by adding 12.5 mL of the plant extract to 25 mL of an aqueous solution of silver nitrate (1.0 × 10^–3^ mol L^–1^). The reaction mixture was maintained under gentle stirring for 15 min at room temperature and protected from light. The pH was adjusted to 7.2 with (0.1 mol L^–1^) NaOH. The formation of AgNP@SA was observed by color change in the solution from light brown to intense yellow, which was confirmed by UV–visible spectroscopy and Dynamic Light Scattering (DLS). The nanoparticles were stored in amber flasks under refrigeration (4 °C) until analysis.

### 2.4 Characterization of Plant Extract and AgNP@SA

The synthesized silver nanoparticles stabilized with *S. adstringens* extract (AgNP@SA) were comprehensively characterized using complementary spectroscopic, microscopic, and physicochemical techniques. The optical properties were initially evaluated by UV–vis using an Agilent HP 8453, operating in the 300–700 nm range. Before analysis, samples were diluted (1:1, v/v) in ultrapure water and sonicated for 2 min. Measurements were performed at room temperature using quartz cuvettes (1 cm path length), with the diluted plant extract serving as a blank. The hydrodynamic diameter, polydispersity index (PdI), and zeta potential were determined via DLS using a Zetasizer Nano ZS ZEN3600, equipped with a 4 mW He–Ne laser (λ = 633 nm) and a backscatter angle of 173°. Samples were prepared under the same dilution and sonication conditions and analyzed at 25 °C using DTS 1070 cuvettes. Data acquisition consisted of 15 runs per measurement with three independent replicates, processed using Zetasizer software (v.7.11).

Fourier Transform Infrared Spectroscopy (FT-IR) was employed to identify functional groups involved in nanoparticle formation and stabilization using a Cary 630 in the range of 4000–400 cm^–1^. To minimize interference from unbound biomolecules, AgNP@SA samples were subjected to two centrifugation cycles (14,000 rpm, 60 min, 25 °C), followed by separation of the supernatant and lyophilization of the pellet, hereafter referred to as AgNP@SA.

The morphology and size of the nanoparticles were investigated using Scanning Transmission Electron Microscopy (STEM) and Transmission Electron Microscopy (TEM). STEM analyses were conducted on a Quanta FEG 250 operating at 200 kV, while TEM images were obtained using a JEOL JEM-2100 under the same accelerating voltage. For both techniques, a drop of the colloidal suspension was deposited onto carbon-coated copper grids and dried at room temperature. Nanoparticle size distribution was further quantified from TEM micrographs using ImageJ software.

Elemental composition and spatial distribution were assessed by Energy-Dispersive X-ray Spectroscopy (EDS) using a Dual Beam Nova 200 Nanolab SEM coupled with an Oxford Instruments detector and AZtec software. Samples were mounted on aluminum stubs with conductive carbon tape, sputter-coated with approximately 5 nm of gold, and analyzed at an accelerating voltage of 15 kV and a working distance of 10 mm.

Ultrahigh-Performance Liquid Chromatography–High Resolution Mass Spectrometry (UHPLC-HRMS/MS) analyses were performed using a system consisting of a Thermo Q Exactive Orbitrap mass spectrometer coupled to a Dionex UltiMate 3000 RSLCnano system. Chromatographic separations were carried out on a C18 column (2.1 mm × 50 mm, 1.7 μm; Waters, Ireland). The mobile phases consisted of water with 0.1% formic acid (A) and acetonitrile with 0.1% formic acid (B), at a flow rate of 250 μL min^–1^. The column temperature was maintained at 40 °C, and the injection volume was 5 μL. The gradient elution program was as follows: 25% B at 0 min; 30% B at 1 min (held for 1 min); 38% B at 3 min; 42% B at 6 min; 60% B at 7 min; 90% B at 11 min (held for 5 min); returned to 25% B between 16–17 min, followed by column re-equilibration at 25% B until 19 min. Mass spectra were acquired in positive-ion mode with a spray voltage of 3.5 kV, capillary temperature of 200 °C, and auxiliary gas heater temperature of 300 °C. Full MS scans were recorded at 70,000 fwhm over an *m*/*z* range of 100–1500, followed by data-dependent MS/MS acquisition at 35,000 fwhm for the five most intense ions per cycle.

Data processing was performed using Xcalibur 4.1 (Thermo Fisher), MZmine 3.9.0, and the GNPS platform, applying a minimum cosine score of 0.70 for spectral annotation. The LC-MS/MS data were analyzed on Feature-Based Molecular Networking (FBMN)^20^ using 2 groups (crude extract and AgNP@SA).

X-ray photoelectron spectroscopy (XPS) measurements were performed at the Brazilian Nanotechnology National Laboratory (LNNano/CNPEM, Campinas, Brazil). The surface atomic composition of the samples was analyzed by XPS (K-Alpha model, Thermo Fisher), equipped with an ultrahigh vacuum chamber operating at 10^–9^ Pa and a monochromated, microfocused, low-power Al Kα X-ray source (1486.6 eV) as the excitation source. Peak fitting of all photoemission spectra was performed using CasaXPS software (version 2.3.14). Three regions of the sample were examined, and the mean values along with the standard deviations were calculated for quantitative analysis.

Finally, Proton Nuclear Magnetic Resonance (^1^H-NMR) spectroscopy was performed to investigate molecular components associated with the extract and nanoparticle system. Lyophilized samples (10 mg) were dissolved in 700 μL of D_2_O (99.9% D), vortexed, and transferred to 5 mm NMR tubes. Spectra were acquired at 25 °C on a Bruker Avance III 600 MHz equipped with a Triple Resonance Inverse Broadband (TBI) probe. Data processing, including baseline correction, alignment, phasing, and referencing, was carried out using MestReNova software (v.12.0.0).

### 2.5 Antimicrobial Activity Assessment

#### 2.5.1 Minimum Inhibitory Concentration (MIC)

The AgNP@SA antimicrobial activity was assessed against *Staphylococcus aureus* (CCT 1485), *Pseudomonas aeruginosa* (CCT 1476), *Enterococcus faecalis* (CCT 1494), and *Escherichia coli* (CCT 1457) strains by broth microdilution in 96-well plates. Bacterial cultures were prepared in LB medium, adjusted to 0.5 on the McFarland scale. Serial dilutions of AgNP@SA (0.54 μg/mL to 51.84 μg/mL) (Table S1, Supporting Information) were tested in Mueller–Hinton broth. After incubation for 24 h at 37 °C, the presence of turbidity was visually verified (Figure S1, Supporting Information).

#### 2.5.2 Minimum Bactericidal Concentration (MBC) Assay

The minimum bactericidal concentration (MBC) was determined after MIC reading: aliquots (10 μL) from wells without visible growth were plated onto MH-agar and incubated for an additional 24 h, and the MBC was defined as the lowest concentration of AgNP@SA capable of eliminating the bacterial population. Growth controls (broth + microorganism) and sterility controls (broth without microorganism) were included. All assays were performed in triplicate for each microorganism and repeated in at least three independent experiments.

### 2.6 Metabolomic Analysis by ^1^H-NMR

Metabolomic analyses were performed following procedures previously described by our group.^21^ Spectra were acquired on a Bruker Avance III 600 MHz spectrometer equipped with a Triple Resonance Inverse Broadband (TBI) probe at 25 °C. Two experimental groups were established using *S. aureus* cultures grown in Brain Heart Infusion (BHI) medium. For both groups, bacterial suspensions of around 1 × 10^7^ CFU were used to prepare polar metabolite extracts. In the control group, eight independent cultures were incubated for 24 h at 37 °C under agitation (200 rpm). In the treated group, eight cultures were incubated for 16 h under the same conditions, after which AgNP@SA was added at a final concentration of 21.6 μg mL^–1^ (corresponding to half of the minimum inhibitory concentration). Incubation was continued for an additional 8 h, totaling 24 h, to match the control group. Bacterial cells were harvested by methanol quenching, centrifuged, and resuspended in 2 mL of chilled phosphate saline buffer (PBS). Cell lysis was performed using an ultrasonic processor for 5 min with 10 s intervals. The resulting lysates were centrifuged at 17,000 × g for 2 min, and the supernatants were lyophilized. Dried extracts were reconstituted in deuterium oxide (D_2_O) for NMR spectral acquisition. The spectra were analyzed by PCA (Principal Component Analysis) and *t* test to identify significantly altered metabolites (*p* < 0.05).

## 3 Results and Discussion

### 3.1 Optical Analysis

The formation of biogenic silver nanoparticles (AgNP@SA) was preliminarily evidenced by a distinct color change in the reaction medium, from the light brown characteristic of the *S. adstringens* extract to an intense yellow. This transition is a well-established indicator of the reduction of Ag^+^ to metallic Ag^0^ and the onset of nanoparticle nucleation. The observed coloration is directly associated with the surface plasmon resonance (SPR) phenomenon, a collective oscillation of conduction electrons typical of metallic nanoparticles, particularly silver, with characteristic absorption in the 400–450 nm range.^22,23^

This mechanism is supported by UV–vis spectrophotometric analysis, which revealed a well-defined absorption band centered at 415 nm, characteristic of the SPR of predominantly spherical AgNP, indicating successful nanoparticle formation with a narrow size distribution and minimal aggregation.^24^ Moreover, the temporal stability of the system, monitored over 90 days, as illustrated in (Figure 1A), showed no significant shift in the SPR peak position, no appreciable band broadening, and only a slight decrease in absorbance intensity, confirming high colloidal stability. These findings demonstrate that metabolites from *S. adstringens* act as effective capping agents, promoting the formation of stable, well-dispersed nanoparticles while preventing aggregation and uncontrolled growth over time.

**Figure 1:**
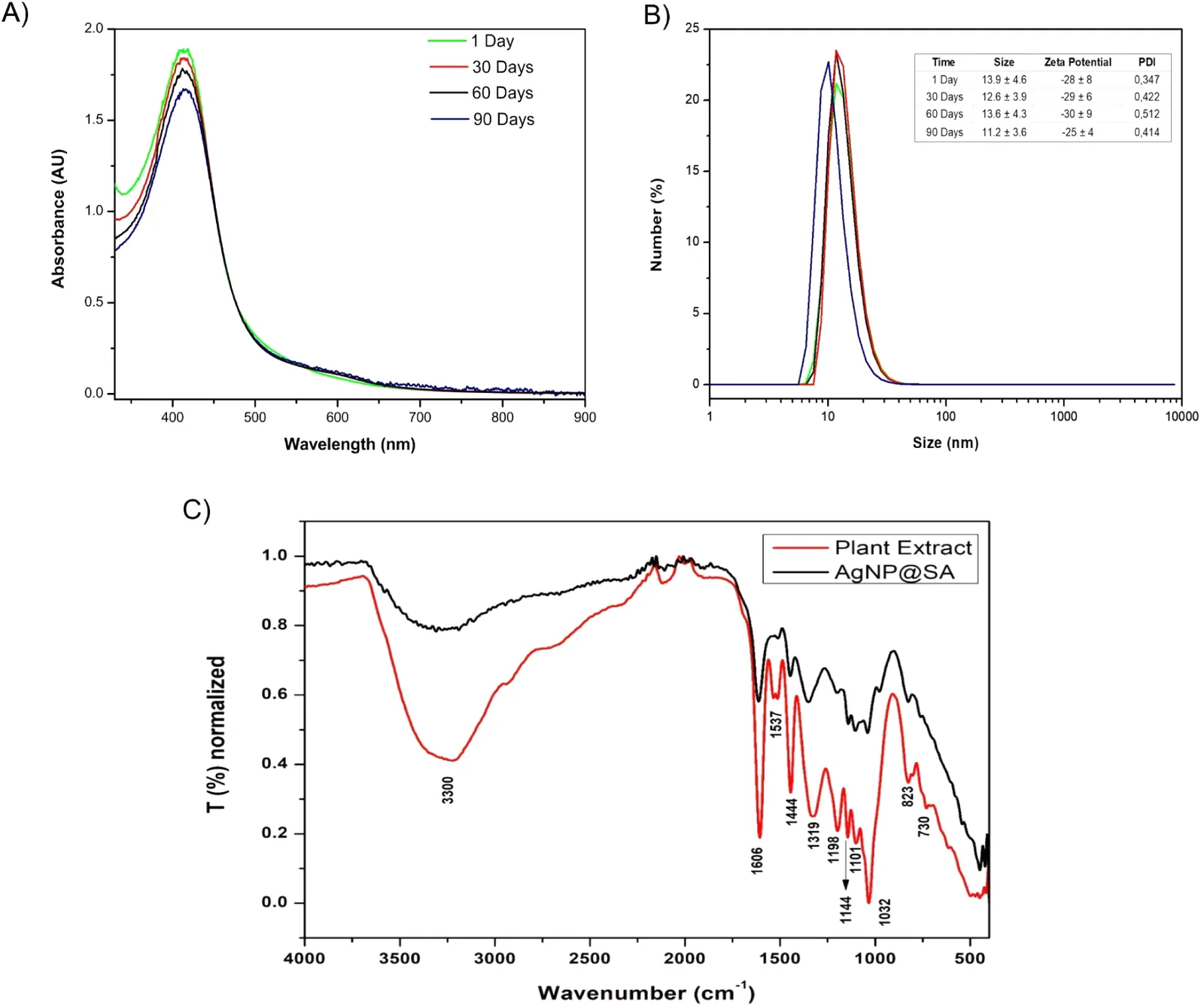
Physicochemical characterization and stability evaluation of biosynthesized silver nanoparticles (AgNP@SA) obtained using *Stryphnodendron adstringens* plant extract. (A) UV–vis absorption spectra of AgNP@SA monitored over 1, 30, 60, and 90 days. (B) Dynamic light scattering (DLS) particle size distribution profiles and corresponding hydrodynamic diameter, zeta potential, and polydispersity index (PDI) values were measured during the storage period. (C) Fourier-transform infrared spectroscopy (FTIR) spectra of the *S. adstringens* plant extract and AgNP@SA.

From a mechanistic perspective, the synthesis of AgNP@SA highlights the efficiency of *S. adstringens* extract as a dual-function system, acting simultaneously as both a reducing and a stabilizing agent. Its high content of polyphenolic compounds, particularly tannins and flavonoids, provides substantial redox potential, enabling the rapid reduction of Ag^+^ ions to metallic Ag^0^. These same biomolecules also adsorb onto the nanoparticle surface, forming a protective organic interface that inhibits uncontrolled growth and aggregation.^22,24^

### 3.2 Dynamic Light Scattering Analysis

The dynamic light scattering (DLS) analysis (Figure 1B) revealed that AgNP@SA exhibited a mean hydrodynamic diameter of 13 ± 4 nm and a polydispersity index (PdI) of 0.4, which is considered acceptable for biosynthesized systems and indicates a relatively homogeneous size distribution. Additionally, the zeta potential was measured at −28 ± 6 mV (Figure S2, Supporting Information), suggesting good colloidal stability, since absolute values above |25| mV are generally associated with sufficient electrostatic repulsion to prevent nanoparticle aggregation.^25,26^ These results underscore the ability of plant-derived metabolites to effectively regulate nanoparticle nucleation and growth. Notably, the close agreement between the hydrodynamic diameter (DLS) and the metallic core size (TEM) suggests the formation of a thin, compact, and nonhydrated organic shell surrounding the silver core. This suggests that the phytochemicals act as a tightly bound capping layer rather than a diffuse corona. Such a structural configuration, combined with the observed spherical morphology and relatively low polydispersity, points to a highly controlled formation process, which is essential for achieving reproducible physicochemical and biological properties. The colloidal stability of AgNP@SA can be further attributed to the electrostatic stabilization provided by the phytochemical layer adsorbed onto the nanoparticle surface. The negative zeta potential reflects the presence of deprotonated functional groups, such as hydroxyl groups from tannins and flavonoids, which generate strong particle electrostatic repulsion, thereby preventing aggregation and ensuring long-term dispersion stability.

### 3.3 Fourier Transform Infrared Spectroscopy (FT-IR) Analyses

Fourier Transform Infrared (FT-IR) spectroscopy was employed to elucidate the functional groups present in the *S. adstringens* extract and to investigate the molecular interactions between its bioactive compounds and the surface of the synthesized AgNP@SA. Analyses were conducted on the crude extract and freeze-dried AgNP@SA, illustrated in (Figure 1C), enabling a comprehensive assessment of the role of plant metabolites in both reduction and stabilization processes. The spectra revealed the presence of characteristic functional groups associated with phenolic compounds and flavonoids across all samples, with prominent bands observed at 3500–3000 cm^–1^ (O–H stretching), 1606 cm^–1^ (C=O), 1444 cm^–1^ (aromatic C=C), 1340 cm^–1^ (C–O–H), and 1032 cm^–1^ (C–H), consistent with hydrolyzable and condensed tannins.^27,28^ Following nanoparticle formation, a noticeable decrease in the intensity of the O–H stretching band, accompanied by the persistence of the C=O band, was observed, suggesting the oxidation of phenolic groups to quinones, thereby confirming the active participation of tannins in the reduction of Ag^+^ ions.^29^ Furthermore, comparison between the spectra of the plant extract and AgNP@SA demonstrated changes in band intensity and broadening, indicating that these biomolecules remain adsorbed onto the nanoparticle surface, acting as stabilizing agents.^30^ This organic capping layer plays a crucial role in ensuring colloidal stability and may significantly influence the biological performance of the nanoparticles, as previously reported for plant-functionalized nanomaterials.^3^ Overall, the FT-IR results corroborate a redox-mediated synthesis mechanism, in which hydroxyl groups from phenolic structures donate electrons for the reduction of Ag^+^ to Ag^0^, followed by their oxidation to quinone species, a pathway widely described for green synthesis approaches.^29^

### 3.4 Scanning Transmission Electron Microscopy (STEM) and Transmission Electron Microscopy (TEM) Analyses

The morphological characterization of the AgNP@SA was performed using Transmission Electron Microscopy (TEM), including scanning mode (STEM). In the present study, the images obtained by STEM (Figure 2A) and TEM (Figure 2B–C) showed nanoparticles with a predominantly spherical morphology, well-defined and without signs of aggregation, indicating colloidal stability. For particle size analysis, the images were processed in ImageJ software, enabling the construction of the size distribution curve (Figure 2D). The average diameter calculated was 12 ± 2 nm, similar to that obtained by DLS (13 ± 4 nm). This discrepancy is characteristic of green-synthesized nanoparticles; while TEM measures the high-electron-density metallic core, DLS accounts for the solvation layer and the phytochemical corona (tannins and flavonoids) adsorbed onto the surface.

**Figure 2:**
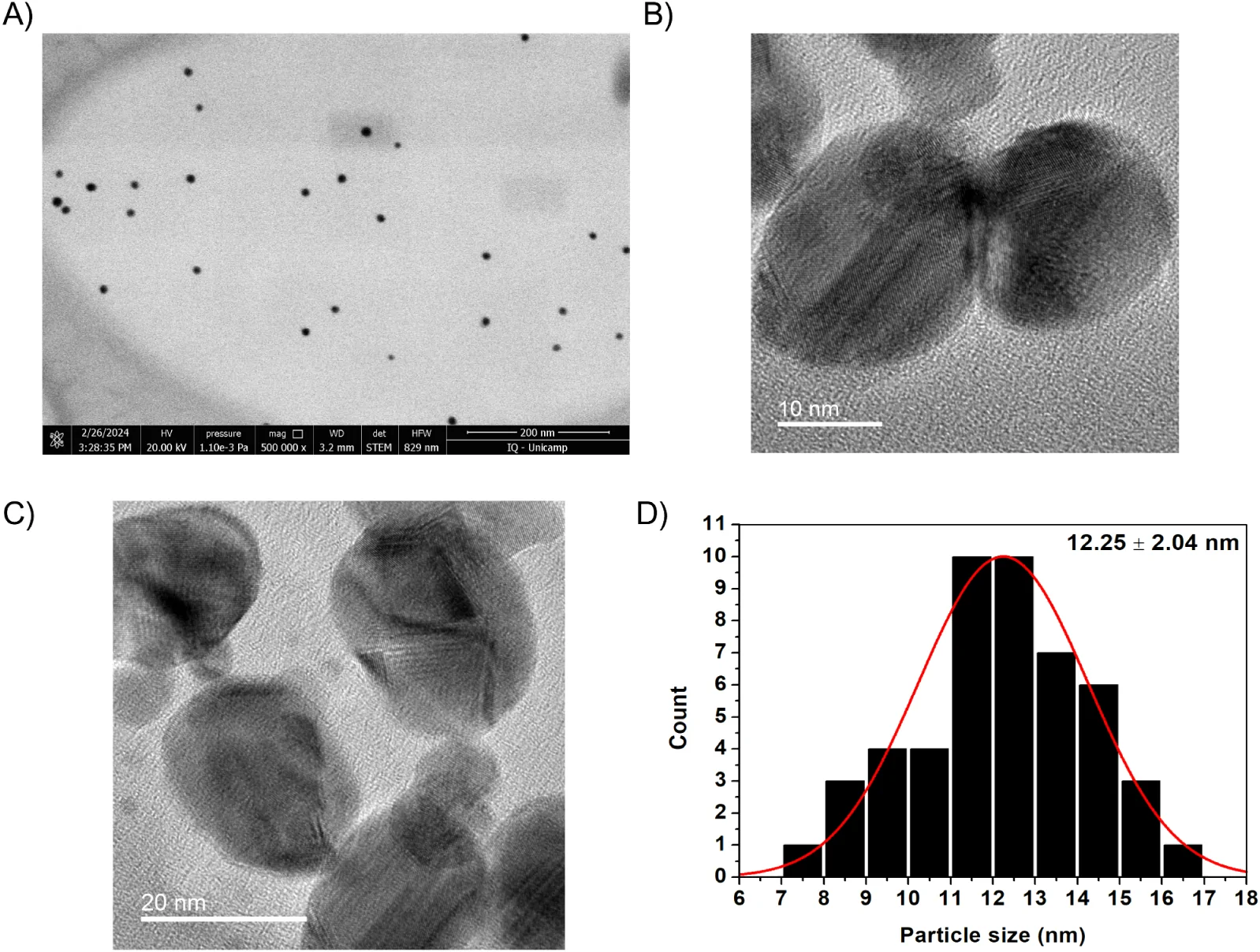
Micrographs of biosynthesized silver nanoparticles (AgNP@SA) produced using *Stryphnodendron adstringens* extract, (A) STEM micrograph of AgNP@SA, showing a homogeneous size distribution and spherical nanoparticle morphology, (B–C) high-resolution TEM images revealing predominantly spherical nanoparticles with well-defined morphology and visible lattice fringes, confirming the crystalline nature of the nanostructures, (D) particle size distribution histogram obtained from TEM image analysis, demonstrating an average particle diameter of 12 ± 2 nm, indicating narrow size distribution and nanoscale uniformity of the biosynthesized AgNP@SA.

### 3.5 Energy-Dispersive X-ray Spectroscopy (EDS) Analyses

The elemental composition of AgNP@SA was analyzed by energy-dispersive X-ray spectroscopy (EDS) coupled with scanning electron microscopy (SEM) (Figure 3A). Elemental mapping (Figure 3B) revealed that silver signals are predominantly localized in regions corresponding to the nanoparticles, confirming the successful formation and distribution of AgNP@SA. The EDS spectrum (Figure 3C) showed an intense carbon peak, indicating the presence of organic material associated with the plant extract, consistent with the adsorption of phytochemicals on the nanoparticle surface. Copper signals were also detected, originating from the copper grid used as a support, while a minor silicon peak may be attributed to contamination, substrate interference, or detector artifacts. Additionally, low-intensity peaks of Na, Al, S, Cl, Ca, and Fe were observed, likely corresponding to trace residues from the synthesis reagents or intrinsic components of the biological extract. Importantly, the detection of carbon provides direct evidence of the organic capping layer, reinforcing the role of plant-derived metabolites in stabilizing the nanoparticles. This naturally formed coating establishes a clear connection between the extract composition and the physicochemical stability of the system, enabling effective colloidal stabilization without the need for synthetic surfactants, which is a significant advantage for potential biomedical and environmental applications requiring high biocompatibility.

**Figure 3:**
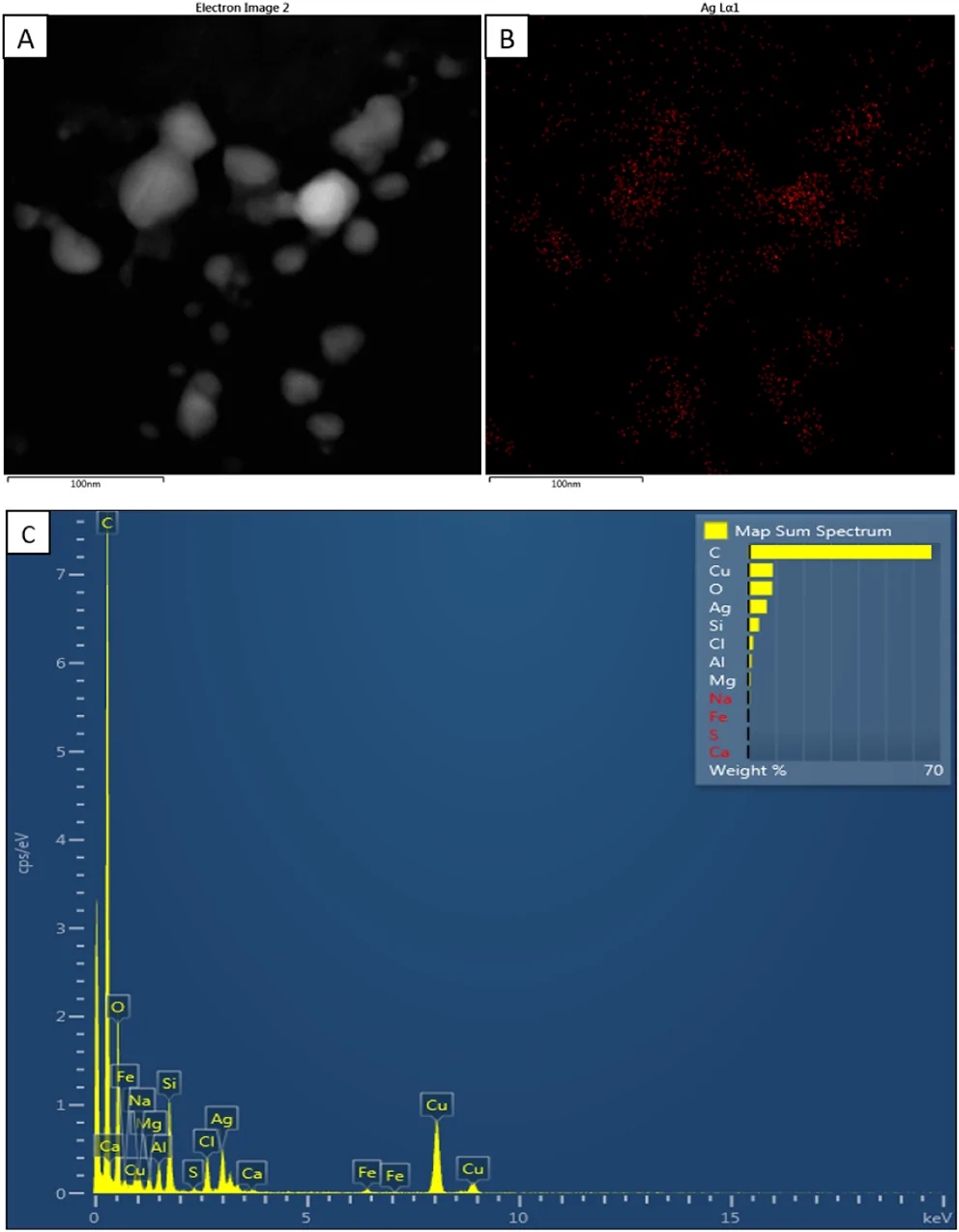
Energy-dispersive X-ray spectroscopy (EDS) image (A) scanning Electron Microscopy (SEM), (B) silver contour within the nanoparticles, and (C) elemental mapping of the electron micrograph region.

### 3.6 XPS Characterization of AgNP@SA Particles

To complement the structural data obtained for AgNP@SA, X-ray photoelectron spectroscopy (XPS) was utilized to evaluate the surface chemistry of the AgNP@SA particles. Quantitative XPS analysis of the AgNP@SA sample revealed the presence of two principal elements, carbon and oxygen. In addition, traces of Ag, S, N, P and Na were detected (Figure 4). The peak deconvolution of the C 1s, O 1s, N 1s, S 2p, P 2p and Ag 3d doublet regions provided detailed insights into the chemical environments involved in nanoparticle formation and stabilization.

**Figure 4:**
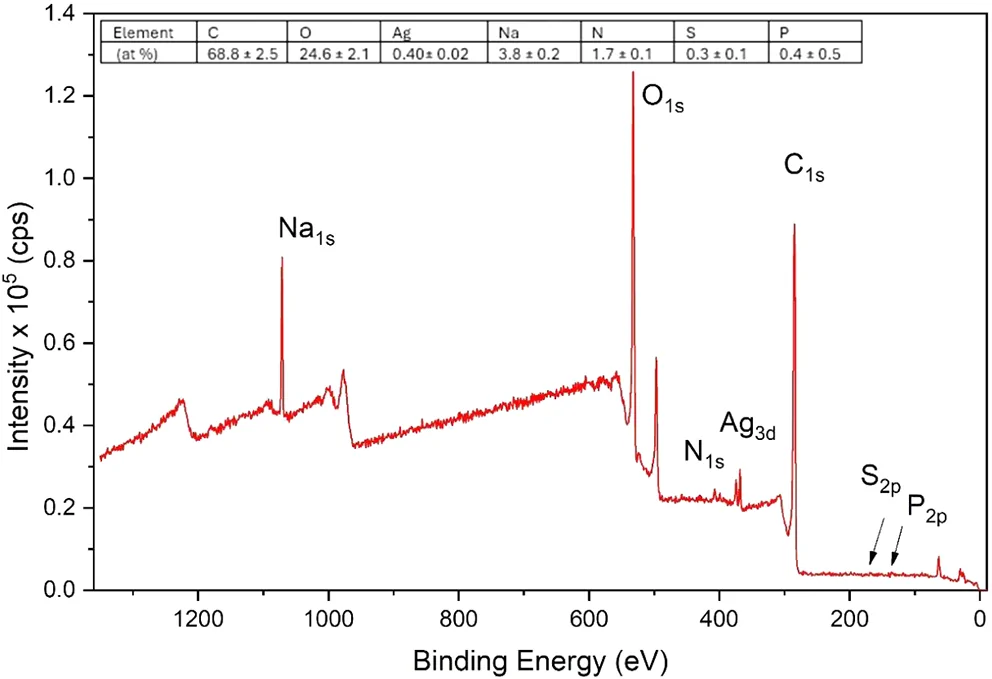
Typical XPS survey spectrum of AgNPs@SA. The principal identified elements are indicated. The table presents quantitative analysis obtained from three regions.

The C 1s peak displayed components at 284.3 eV, corresponding to aromatic C–C/C=C from flavonoid and tannin backbones, at 286.0 eV, assigned to C–O groups from phenolic hydroxyls and flavonoids, and at 286.8 eV, attributed to C=O/O–C–O functionalities from tannins and oxidized carbonyls^31,32^ (Figure 5A). These signals are consistent with FT-IR evidence showing the oxidation of phenolic O–H groups to quinones and the persistence of carbonyl bands, confirming the active participation of tannins in the reduction of Ag^+^ ions. The O 1s envelope revealed components at 530.8 eV, indicative of Ag–O bonds,^33^ at 532.7 eV, corresponding to C–O groups,^34,35^ and at 535.1 eV, related to adsorbed water or oxidized oxygen species,^36^ corroborating the role of hydroxyl groups in electron donation and surface coordination (Figure 5B).

**Figure 5:**
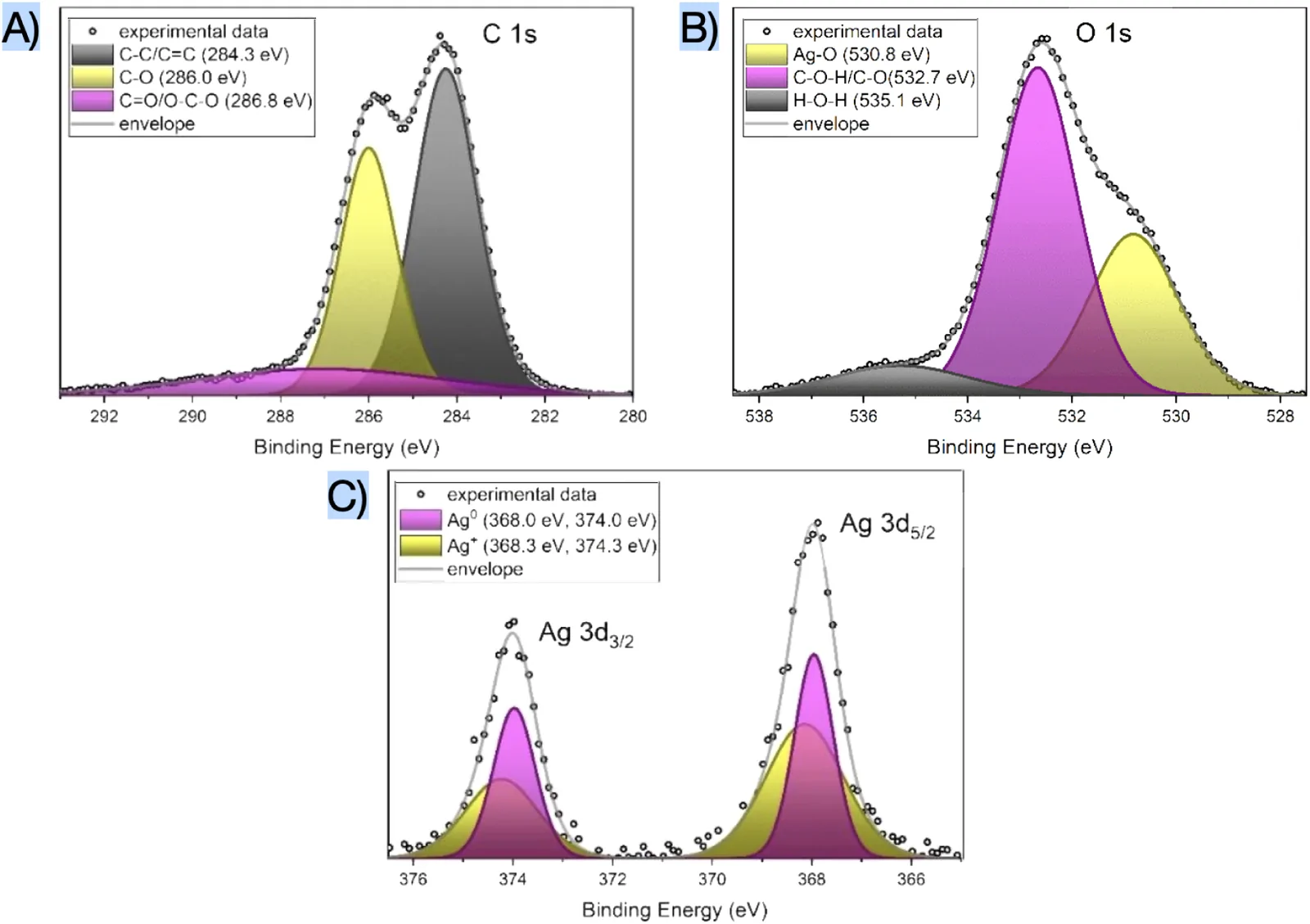
High-resolution XPS spectra (A) C 1s, (B) O 1s, and (C) doublet of the Ag 3d lines obtained from the AgNP@SA sample. The typical examples of deconvolutions are shown. Component peak positions and the possible identifications are indicated in the legends.

Deconvolution of the Ag 3d doublet yielded components at 368.0/374.0 eV, corresponding to metallic silver (Ag^0^),^37^ and at 368.3/374.3 eV (both with 6 eV of spin–orbit splitting), assigned to oxidized silver (Ag^+^)^38–40^ coordinated to organic ligands, confirming the coexistence of reduced cores and oxidized surface species typical of green synthesis (Figure 5C). The N 1s signals at 399.4 and 407.2 eV were attributed to aromatic or amide nitrogen coordinated to silver and oxidized nitrogen species (NO_2_/NO_3_),^41,42^ respectively (Figure S3, Supporting Information). The S 2p peak at 168.6 eV indicated oxidized sulfur functionalities,^43,44^ which can be related to sulfate or sulfonated groups originating from plant metabolites (Figure S3, Supporting Information). Additionally, the Na 1s signal at 1071.4 eV (Figure S3, Supporting Information) reflected residual sodium introduced during pH adjustment with NaOH (e.g., NaNO_3_),^45^ contributing to ionic balance and colloidal stability. Finally, the peaks at 135.6 and 133.7 eV in the P 2p region (Figure S3, Supporting Information) indicated the presence of oxidized phosphorus species,^46^ possibly phosphate groups introduced from the plant extract or residual salts, which can also contribute to surface functionalization and stabilization. Together with the Na 1s signal previously observed at 1071.4 eV, reflecting residual sodium from pH adjustment, and FT-IR data, showing oxidation of phenolic O–H groups to quinones and persistence of C=O bands, these results confirm a redox-mediated synthesis mechanism in which phenolic hydroxyls donate electrons to reduce Ag^+^ to Ag^0^, while oxidized metabolites—including carbonyl, nitrogenous, sulfur, and phosphorus functionalities—remain adsorbed as a stabilizing organic capping layer.

### 3.7 Metabolite Annotation by LC-HRMS/MS

The GNPS 2 platform was employed for compound annotation using feature-based molecular networking (FBMN), resulting in the identification of six phenolic compounds (Table 1). Representative mirror spectra of the annotated features are provided in the Supporting Information, Figures S4–S9. Untargeted metabolomic profiling revealed a strong phenolic and flavonoid signature, reflecting the diversity of plant-derived secondary metabolites present in the extract.

**Table 1:** Annotated Compounds using the Database GNPS 2

N°	*m*/*z* experimental	RT (min) plant extract	RT (min) nanoparticle	Annotation	Molecular formula
1	273.076	2.80	2.72	Naringenin	C_15_H_12_O_5_
2	185.081	1.07	1.02	3,4,5-Trimethoxyphenol	C_9_H_12_O_4_
3	307.081	0.60	-	Epigallocatechin	C_15_H_14_O_7_
4	277.071	0.99	0.97	Teadenol A	C_14_H_12_O_6_
5	257.081	1.90	1.81	Isoliquiritigenin	C_15_H_12_O_4_
6	239.091	2.20	2.13	3,4,5-Trimethoxycinnamic acid	C_12_H_14_O_5_

The annotated compounds include key flavonoid classes and their biosynthetic precursors, such as the flavanol (−)-epigallocatechin, the flavanone naringenin, and the chalcone isoliquiritigenin. In addition, phenylpropanoid derivatives, including 3,4,5-trimethoxycinnamic acid, as well as the biotransformed phenolic compound teadenol A, were detected, collectively highlighting a chemically rich and structurally diverse polyphenolic profile.

Comparative analysis between the crude extract of *S. adstringens* and the biosynthesized silver nanoparticles (AgNP@SA) revealed distinct metabolic profiles, providing insights into the mechanisms underlying the green synthesis process. The absence of (−)-epigallocatechin in the nanoparticle sample strongly suggests its involvement as a primary reducing agent. During synthesis, the abundant phenolic hydroxyl groups of this flavonoid likely donate electrons to reduce Ag^+^ to metallic Ag^0^, undergoing concomitant oxidation to quinone-like structures. Consequently, its consumption explains its disappearance from the mass spectrometric profile of AgNP@SA.

Molecular networking analysis using GNPS further corroborates these metabolic changes. The node corresponding to (−)-epigallocatechin (*m*/*z* 307.081), along with related nodes within the same molecular cluster, is exclusively detected in the crude extract (pink nodes), and is absent in the AgNP@SA sample (Figure 6A). Given that GNPS clusters features based on structural similarity, the disappearance of this entire cluster suggests that the reduction of Ag^+^ ions was not mediated by a single compound, but rather by a synergistic pool of structurally related polyphenols, which were collectively oxidized and consumed during nanoparticle formation.

**Figure 6:**
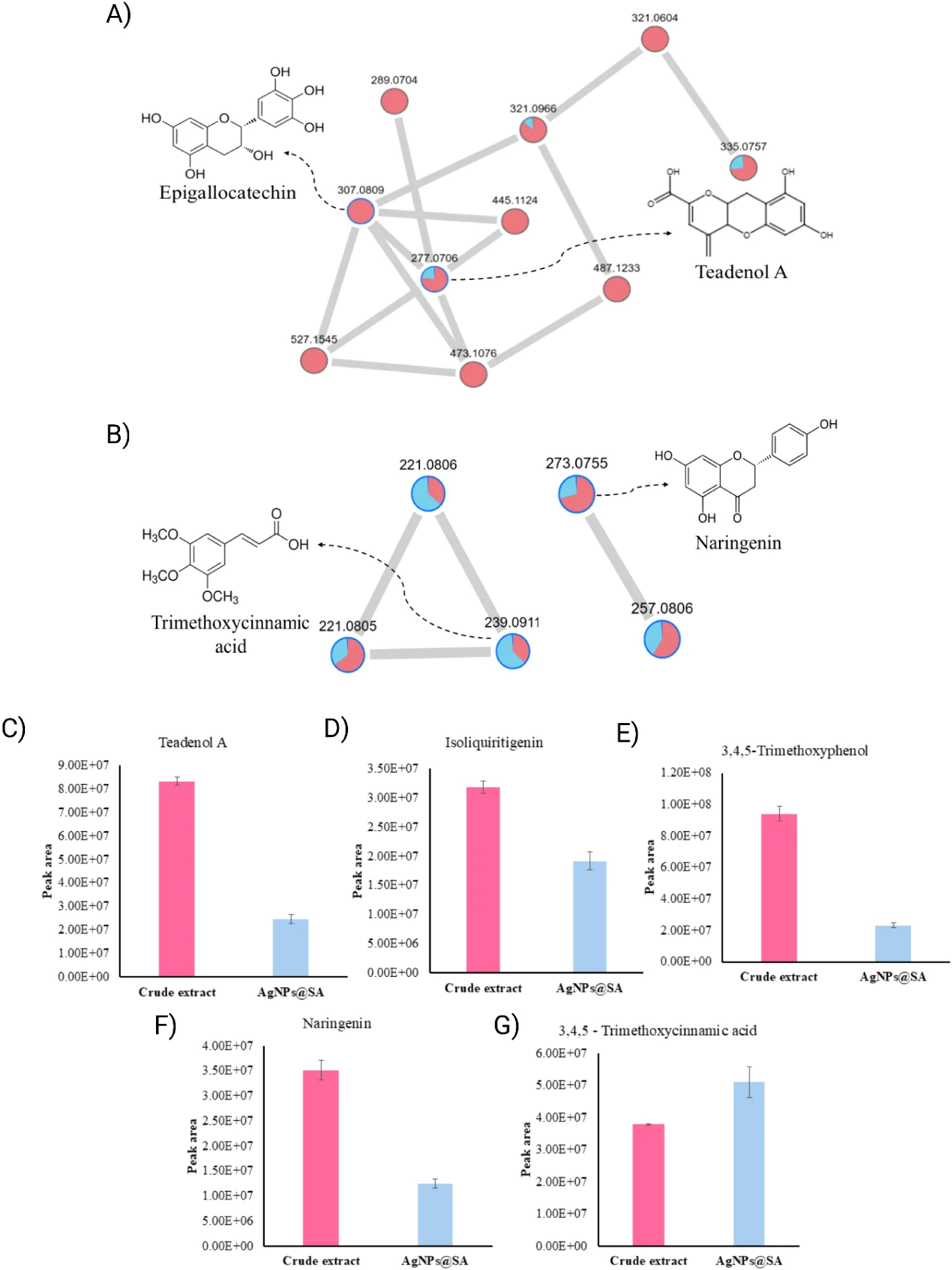
Molecular networking analysis generated by GNPS: Crude extract is represented in pink color, and AgNP@SA in blue color. Each node shows *m*/*z* values. (A) Molecular cluster of the compounds related to epigallocatechin (*m*/*z* 307.081). (B) Molecular cluster of the phenolic acid and flavanones. (C–G) Compound distribution between the samples according to the peak area of the chromatogram.

Beyond the reduction process, the metabolomic profile also highlights a complementary stabilization mechanism. Compounds such as 3,4,5-trimethoxycinnamic acid and naringenin are likely involved as capping agents (Figure 6B). Their functional groups, including phenolic hydroxyls, methoxy, and carboxyl moieties, exhibit strong affinity for the silver surface, enabling coordination and formation of a stabilizing organic corona. This interfacial layer provides steric and electrostatic stabilization, preventing aggregation and helping maintain nanoscale dimensions. Overall, the data show that *S. adstringens* extract operates in a dual synergistic role, simultaneously triggering the reduction of silver ions and ensuring the stabilization of the resulting nanoparticles through a complex phytochemical network.

A notable chromatographic phenomenon observed in this study was a consistent shift in retention times (RT) of several phenolic and flavonoid compounds. All metabolites, including teadenol A (0.99 → 0.97 min), 3,4,5-trimethoxyphenol (1.07 → 1.02 min), isoliquiritigenin (1.90 → 1.81 min), and 3,4,5-trimethoxycinnamic acid (2.20 → 2.13 min), exhibited earlier elution in the AgNP@SA sample compared to the crude extract. This systematic decrease in RT supports the involvement of these compounds as capping agents. Such shifts are likely associated with transient complexation between electron-rich phytochemicals and the nanoparticle surface, which alters their effective polarity and partitioning behavior. In addition, matrix effects arising from the colloidal nanoparticle environment may further reduce interactions with the stationary phase, collectively resulting in decreased retention times. Together, these observations provide indirect but consistent chromatographic evidence of phytochemical adsorption onto the silver nanoparticle surface.

### 3.8 Nuclear Magnetic Resonance Spectroscopy ^1^H-NMR

The ^1^H-NMR spectra (Figure 7) corroborate the FT-IR and LC-HRMS/MS results, confirming the rich polyphenolic composition of the *S. adstringens* extract. Peaks at 7.06 ppm, attributed to gallic acid and hydrolyzable tannins, together with resonances in the 5.8–7.3 ppm region corresponding to flavonoid aromatic protons, were clearly observed. Additional peaks between 2.8 and 4.7 ppm are consistent with flavan-3-ol units, characteristic of condensed tannins.^47^ These assignments collectively confirm the presence of both hydrolyzable and condensed tannins, which are well-established major constituents of *S. adstringens*.^27,28^ Following nanoparticle synthesis (AgNP@SA), notable spectral changes were observed, including signal broadening and a decrease in peak intensity. In particular, the attenuation and partial disappearance of resonances in the 5.9–6.9 ppm region reveal the direct involvement of aromatic polyphenols in nanoparticle formation. This spectral region is typically associated with aromatic protons of tannins and related phenolic compounds, suggesting their active participation in Ag^+^ reduction and Ag^0^ surface stabilization. The observed changes are consistent with partial oxidation of phenolics during metal ion reduction and/or strong coordination of these molecules to the silver surface as capping agents. Such interactions reduce molecular mobility and alter relaxation properties, resulting in significant line broadening and signal suppression in solution-state NMR. These findings strongly support the role of tannins and other phenolic constituents as both reducing and stabilizing agents, leading to the formation of a phytochemically functionalized nanoparticle interface. This organic corona enhances colloidal stability by preventing aggregation, a common limitation in nanoparticle synthesis.^29^

**Figure 7:**
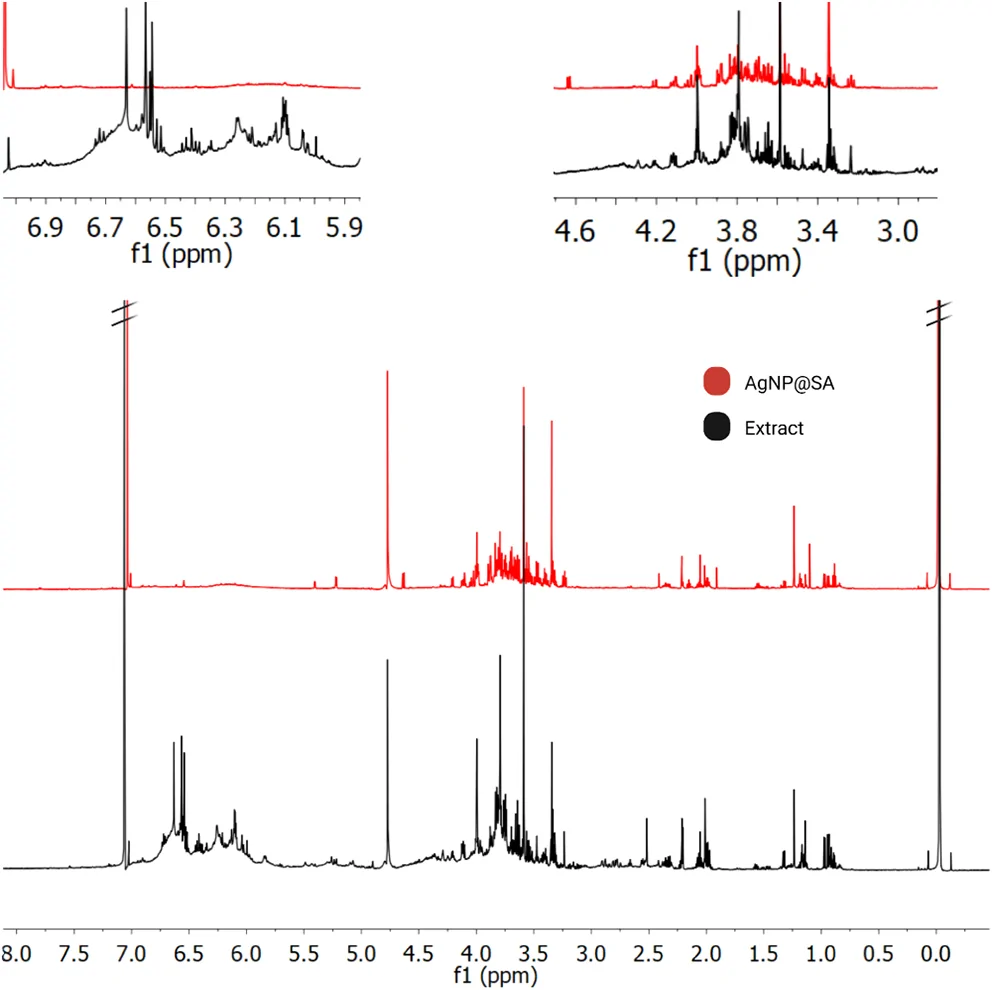
^1^H-NMR spectra: overlap of the spectra of the aqueous extract of *S. adstringens* bark (black line) and AgNP@SA (red line).

To facilitate the interpretation of the green synthesis mechanism, the major metabolites identified in the *S. adstringens* bark extract are illustrated in Figure 8A. Polyphenolic constituents, particularly tannins and flavonoid derivatives, possess multiple phenolic hydroxyl groups capable of donating electrons to Ag^+^ ions, promoting their reduction to metallic silver (Ag^0^) (Figure 8B). Following nucleation, these compounds adsorb onto the nanoparticle surface through coordination of oxygen atoms and hydrogen-bonding interactions, acting as natural capping agents that prevent aggregation and contribute to the observed colloidal stability.^48–50^

**Figure 8:**
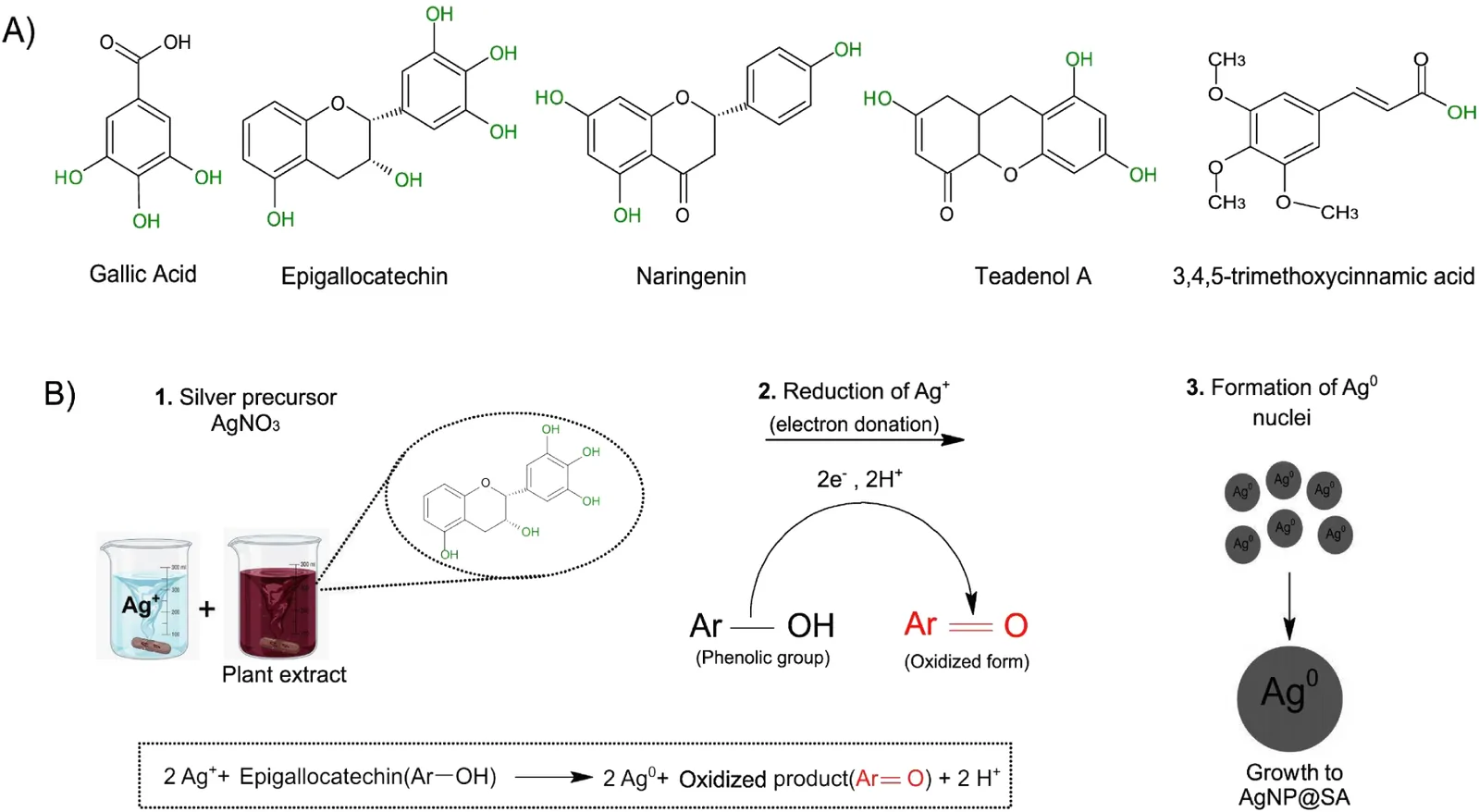
Proposed mechanism for the green synthesis of AgNP@SA using *S. adstringens* bark extract. (A) Representative phytochemicals identified in the *S. adstringens* bark extract by UHPLC-HRMS/MS and ^1^H-NMR, epigallocatechin was detected in the extract but was absent from the AgNP@SA system, suggesting its consumption during the reduction of Ag^+^ ions. (B) Proposed mechanism for AgNP@SA formation, in which polyphenolic compounds reduce Ag^+^ to Ag^0^ through oxidation of phenolic hydroxyl groups, followed by nucleation and nanoparticle growth.

## 4 Antimicrobial Activity

The biosynthesized silver nanoparticles exhibited significant antimicrobial activity against all tested strains, with MIC values ranging from 17–35 μg mL^–1^ and MBC values from 26–43 μg mL^–1^ (Table 2). From a biotechnological perspective, this activity is closely linked to the phytochemical capping layer derived from *S. adstringens*, which plays a central role in modulating nanoparticle–microorganism interactions. Unlike conventionally synthesized nanoparticles, AgNP@SA are functionalized with tannins and flavonoids, forming a bioactive interface that not only stabilizes the nanostructure but also enhances antimicrobial performance while potentially reducing host cytotoxicity.^3,21^ This dual functionality underscores a key advantage of green nanotechnology, in which the same metabolites responsible for nanoparticle formation also contribute synergistically to their biological activity.

**Table 2:** Identification of Microorganisms Tested and Their Respective Minimum Inhibitory Concentrations and Minimum Bactericidal Concentrations

Strains	Origin	Group	MIC	MBC
*P. aeruginosa*	*CCT 1476*	Gram-negative	35 μg mL^–1^	43 μg mL^–1^
*E. coli*	CCT 1457	Gram-negative	35 μg mL^–1^	43 μg mL^–1^
*S. aureus*	CCT 1457	Gram-positive	43 μg mL^–1^	52 μg mL^–1^
*Ent. faecalis*	CCT 1494	Gram-positive	17 μg mL^–1^	26 μg mL^–1^

Interestingly, although silver nanoparticles typically display higher efficacy against Gram-negative bacteria due to their thinner peptidoglycan layer, the present results deviate from this trend, with the lowest MIC observed against *E. faecalis faecali*, a Gram-positive organism, indicating increased susceptibility. This enhanced sensitivity may be attributed to synergistic interactions between the phytochemical coating and structural components of Gram-positive cell walls. As reported by Hamouda et al. (2023),^51^ plant-derived metabolites can modulate nanoparticle activity by facilitating penetration and enhancing antimicrobial effects across diverse microbial systems. In particular, phenolics, flavonoids, and terpenoids may interact with teichoic and lipoteichoic acids, increasing membrane permeability and promoting nanoparticle uptake.^52^ Thus, the phytochemical corona acts not merely as a stabilizing shell but as an active functional layer that amplifies the antimicrobial efficacy of the silver core. This interpretation aligns with recent evidence highlighting capping agents as key determinants of the biological performance of biogenic nanoparticles, where specific coatings, such as saponin- or polyphenol-rich layers, can destabilize microbial membranes and enhance bactericidal effects.^20^ Furthermore, the strong activity against *E. faecalis*, including drug-resistant strains, is consistent with studies showing that phytochemically functionalized AgNPs can disrupt membrane potential and inhibit biofilm formation.^53,54^ Collectively, these findings reinforce the potential of AgNP@SA as effective antimicrobial agents within a sustainable green synthesis framework.

The literature demonstrates that both *S. adstringens* extract and silver nanoparticles (AgNP@SA) exhibit antimicrobial activity, although through distinct mechanisms. The extract exerts its antimicrobial effects mainly due to its high content of tannins and phenolic compounds, which promote protein precipitation and disrupt bacterial cell wall integrity and membrane permeability. However, this activity generally requires relatively high concentrations and exhibits a narrower antimicrobial spectrum than silver nanoparticles.^14,15^ In contrast, conventionally synthesized AgNPs display potent bactericidal activity through the release of Ag^+^ ions, generation of reactive oxygen species (ROS), and damage to bacterial proteins, membranes, and DNA, although they often exhibit lower colloidal stability and greater cytotoxic potential.^1–3^ Conversely, AgNP@SA combine the intrinsic antimicrobial mechanisms of the metallic silver core with the bioactivity of phenolic metabolites adsorbed onto their surface, creating a functional phytochemical interface that enhances interactions with bacterial cells. Therefore, although the present study did not experimentally compare chemically synthesized AgNP and the *S. adstringens* extract alone, the obtained results, together with evidence from the literature, suggest that the natural functionalization provided by *S. adstringens* metabolites in AgNP@SA represents a significant advantage over conventional AgNP by improving colloidal stability, promoting potential synergistic antimicrobial effects, and providing a more favorable biocompatibility profile, all of which are highly desirable features for future biomedical applications.

## 5 Metabolomic Analysis

The impact of silver nanoparticles synthesized using *S. adstringens* extract (AgNP@SA) on the metabolic profile of *S. aureus* was investigated by ^1^H-NMR spectroscopy, revealing pronounced biochemical alterations upon exposure. A wide range of metabolites was identified (Figure 9A), and PCA showed a clear separation between treated and control groups (Figure 9B). The scores plot demonstrated distinct clustering primarily along PC 1 (59.8% of variance), while PC 2 accounted for 20% of variance, resulting in a cumulative variance of 79.8%. This strong segregation supports the occurrence of substantial treatment-induced metabolic reprogramming. The loadings plot (Figure 9C) identified the key metabolites driving this discrimination. Acetate, betaine, and lysine showed strong negative loadings on PC 1, correlating with the AgNP@SA-treated group, whereas the branched-chain amino acids (BCAAs), leucine and valine, were associated with the control group. Quantitative analysis confirmed significant decreases in leucine (Figure 9D) and valine (Figure 9E) levels (*p* < 0.05), alongside a marked increase in lysine (Figure 9F) (*p* < 0.01). In addition, acetate (Figure 9G) and betaine (Figure 9H) were significantly elevated in treated samples (*p* < 0.05), collectively evidencing extensive metabolic disturbance. From a mechanistic perspective, these alterations indicate that AgNP@SA interfere with essential metabolic networks in *S. aureus*. The reduction in leucine and valine levels confirms an alteration in BCAA metabolism, which is fundamental for protein biosynthesis and energy homeostasis.^55^ Consistent with previously reported silver nanoparticle-induced stress mechanisms, including membrane damage, enzymatic inhibition, and impaired DNA replication.^56^ The increase in lysine may reflect metabolic dysregulation or a compensatory stress response, further supporting global metabolic imbalance. Elevated acetate levels suggest a shift in central carbon metabolism toward fermentative pathways, likely as an adaptive response to nanoparticle-induced stress.^57^ Similarly, increased betaine, a well-known osmoprotectant, confirms activation of cellular defense mechanisms against oxidative stress and membrane perturbation.^58,59^ Together, these results demonstrate that AgNP@SA not only exert potent antimicrobial activity but also induce deep metabolic reprogramming in *S. aureus*, reinforcing their potential as multifunctional antimicrobial agents within a sustainable nanotechnology framework.

**Figure 9:**
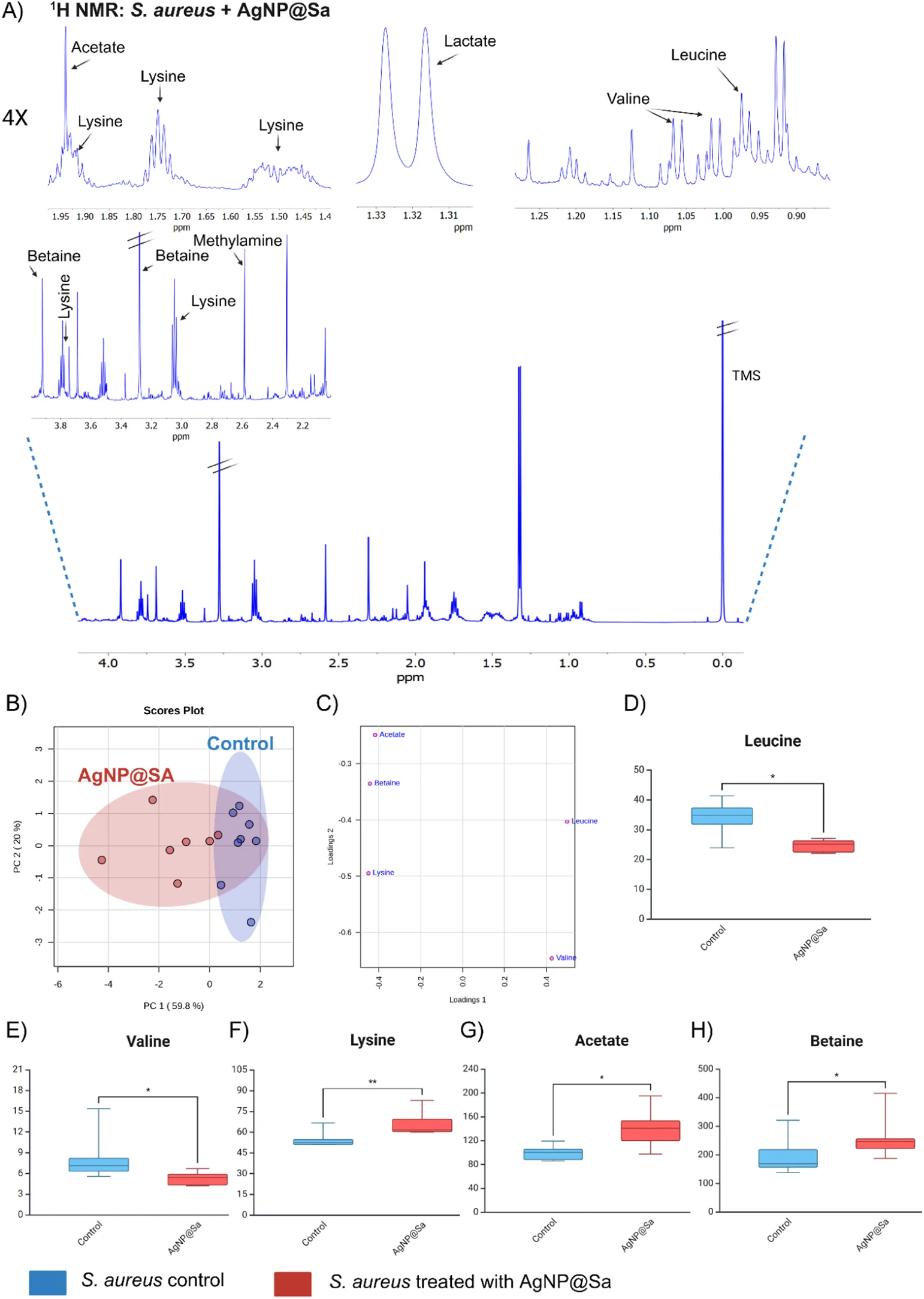
^1^H-NMR-based metabolomics on *S. aureus* exposed to the biosynthesized silver nanoparticles (AgNP@SA): (A) representative ^1^H-NMR spectra highlighting the main metabolites identified after treatment with AgNP@SA, including acetate, lysine, lactate, valine, leucine, betaine, and methylamine, which suggests alterations in bacterial metabolic pathways. (B) PCA scores plot demonstrating clear metabolic discrimination between the control group and *S. aureus* treated with AgNP@SA. (C) Loadings plot showing the metabolites responsible for group separation in the multivariate analysis. (D–H) Boxplot analysis of significantly altered metabolites, including leucine, valine, lysine, acetate, and betaine, evidencing metabolic perturbations associated with AgNP@SA exposure. Statistical significance is indicated by asterisks (*p < 0.05; **p < 0.01).

Although the release of Ag^+^ ions was not directly evaluated in this study, it is widely recognized that partial oxidation of AgNPs under aqueous and physiological conditions can generate dissolved silver­(I) ions, which contribute to their antimicrobial activity.^60,61^ Ag^+^ ions readily interact with thiol-containing proteins, membrane components, and nucleic acids, disrupting essential cellular functions and promoting bacterial cell death.^62,63^ Nevertheless, increasing evidence confirms that the antimicrobial activity of AgNPs cannot be attributed solely to ion release. Direct interactions between nanoparticles and bacterial membranes, the induction of oxidative stress, membrane damage, and intracellular metabolic perturbations also play essential roles.^64–66^ In the present study, the significant alterations observed in the metabolomic profile of *S. aureus* are the result of metabolic stress and disruption of essential cellular pathways. These metabolic changes are consistent with a multifactorial mechanism of action involving nanoparticle–cell interactions, oxidative stress, and impairment of bacterial metabolism.^67–69^ Therefore, while a contribution from Ag^+^ ions cannot be excluded, the combined antimicrobial and metabolomic findings suggest that the bactericidal activity of AgNP@SA results from the synergistic action of nanoparticle-mediated effects and the possible release of silver ions, rather than from Ag^+^ release alone.

## 6 Sustainability Assessment

The sustainability assessment, based on the 12 Principles of Green Chemistry, shows that the synthesis route is highly efficient and environmentally benign. The process achieves near 100% atom economy for silver, produces no toxic byproducts, and exhibits a low E-factor compared to conventional methods that rely on organic solvents and synthetic stabilizers such as PVP. Waste generation is minimal, consisting mainly of water and biodegradable plant residues, resulting in a reduced environmental footprint. Additionally, the use of *Stryphnodendron adstringens* extract replaces hazardous reducing agents such as sodium borohydride (NaBH_4_), employing a renewable and biocompatible alternative. The reaction proceeds under mild conditions (25 °C, atmospheric pressure), eliminating energy-intensive steps, while ultrapure water serves as the sole solvent. Together, these features highlight a simple, energy-efficient, and sustainable nanofabrication strategy aligned with green nanotechnology principles.

## 7 Conclusion

In this study, a sustainable green synthesis strategy was successfully developed for the production of silver nanoparticles using *Stryphnodendron adstringens* extract (AgNP@SA), yielding highly monodisperse spherical nanostructures with an average diameter of 12 ± 2 nm and remarkable colloidal stability exceeding 90 days. This stability is attributed to the dual role of tannins and polyphenolic compounds acting as both reducing and capping agents. Comprehensive spectroscopic and mass spectrometric analyses (UV–vis, ^1^H-NMR, XPS and UHPLC-MS/MS) confirmed the active participation of these phytochemicals in nanoparticle formation and stabilization. The AgNP@SA displayed broad-spectrum antibacterial activity, with the highest efficacy observed against*Enterococcus faecalis* (MIC = 17 μg mL^–1^). Metabolomic profiling by ^1^H-NMR further revealed that antimicrobial effects are associated with pronounced metabolic dysregulation, particularly disruption of branched-chain amino acid pathways and activation of oxidative stress-related responses, indicating a multifactorial mechanism of bacterial toxicity. Overall, the synergistic interaction between the bioactive plant matrix and the metallic core highlights AgNP@SA as a promising nanobiotic for advanced antimicrobial applications. However, before clinical translation, further studies are required to evaluate cytotoxicity, hemocompatibility, and silver ion release kinetics in relevant *in vivo* models, ensuring their safety, stability, and therapeutic reliability as next-generation nanoantimicrobial agents.

## Supplementary Material



## Data Availability

All experimental data supporting the findings of this study, as well as the experimental procedures, are available within this article. Raw data are available from the corresponding author upon reasonable request.

## Notes

Table S1 and Figures S1 and S2: the antibacterial activity and colloidal stability of the synthesized nanomaterials, respectively; Figure S3: the N 1s, S 2p_3/2_, Na 1s, and P 2p lines from the XPS spectra of AgNPs@SA; Figures S4, S5, S6, S7, S8, and S9: the mass spectra for the compounds naringenin, 3,4,5-trimethoxyphenol, epigallocatechin, teadenol A, isoliquiritigenin, and 3,4,5-trimethoxycinnamic acid, respectively (PDF)

## #

Farroupilha Federal Institute of Education, Science and Technology, Alegrete Campus, 97050–685, Santa Maria, RS, Brazil
