# Supplementary material for: Silver Nanoparticles from Stryphnodendron adstringens: A Green Synthesis Approach, Characterization, and Their Impact on Bacterial Metabolism
Source: ACS Omega. 2026 Aug 14;11(34):51770–83. doi: 10.1021/acsomega.6c06132 (PMC13625129; doi:10.1021/acsomega.6c06132)
Supplement: Supplementary file 1 [file ao6c06132_si_001.pdf]

Silver nanoparticles from *Stryphnodendron adstringens*: A green synthesis approach, characterization, and their impact on bacterial metabolism

[1,2]Maelson Cardoso Lacerda, [1,2]Thyerre Santana da Costa, [1,2]Uedson da Silva das Neves, [1,2,#]Symone Costa de Castro, [3]Andrei Alaferdov, [3]Raluca Savu, [1,2*]Ljubica Tasic

[1]Laboratory of Biological Chemistry (LQB), Institute of Chemistry, and [2] National Institute of Science and Technology of Bioanalytics – Lauro Kubota (INCT-Bio L.K.), Universidade Estadual de Campinas (UNICAMP), 13083-970, Campinas–SP, Brasil

[3]Center for Semiconductor Components and Nanotechnologies (CCS Nano), Universidade Estadual de Campinas (UNICAMP), 13083-870, Campinas-SP, Brazil

*Corresponding author:

E-mail: ljubica@unicamp.br - Ljubica Tasic.

Table S1 - Volumes for microdilution of AgNP@SA, placed in each well of the plate, for MIC testing

Compositions of AgNP@SA dilutions	1st Well - Positive control (C+)	100 µL medium + 100 µL inoculum
	2nd Well - Negative control (C-)	100 µL medium + 100 µL AgNP@SA
	3rd Well	50 µL medium + 150 µL AgNP@SA + 50 µL inoculum
	4th Well	75 µL medium + 125 µL AgNP@SA + 50 µL inoculum
	5th Well	100 µL medium + 100 µL AgNP@SA + 50 µL inoculum
	6th Well	125 µL medium + 75 µL AgNP@SA + 50 µL inoculum
	7th Well	150 µL medium + 50 µL AgNP@SA + 50 µL inoculum
	8th Well	175 µL medium + 25 µL AgNP@SA + 50 µL inoculum
	9th Well	187.5 µL medium + 12.5 µL AgNP@SA + 50 µL inoculum
	10th Well	193.5 µL medium + 6.25 µL AgNP@SA + 50 µL inoculum
	11th Well	196.75 µL medium + 3.125 µL AgNP@SA + 50 µL inoculum
	12th Well	198.44 µL medium + 1.56 µL AgNP@SA + 50 µL inoculum

[#]Farroupilha Federal Institute of Education, Science and Technology, Alegrete Campus, 97050-685, Santa Maria, RS, Brazil

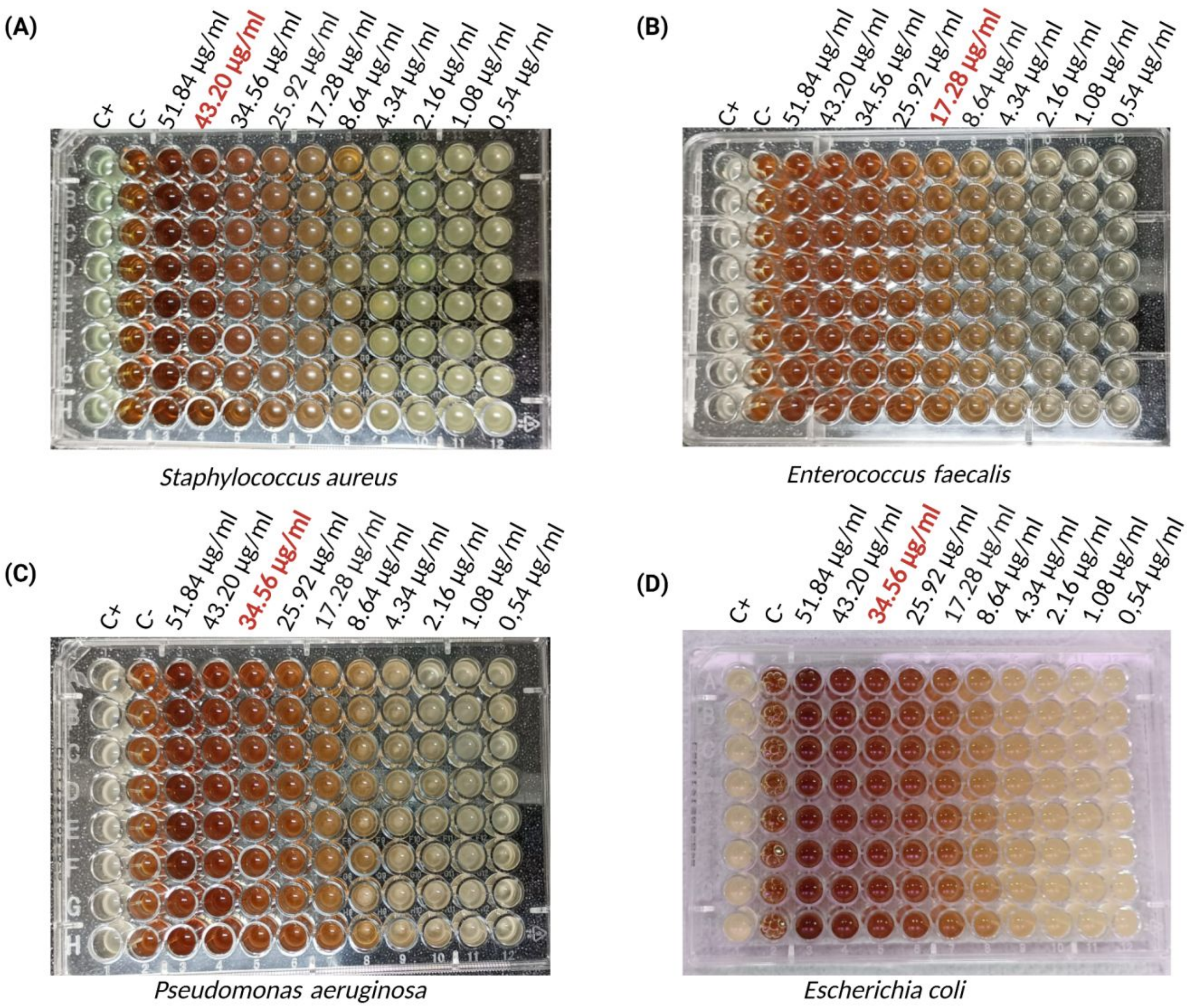

Fig. S1 - 96-well plates with different concentrations of AgNP@SA after 24 h of incubation: **(A)** *Staphylococcus aureus* (CCT1485), **(B)** *Enterococcus faecalis* (CCT 1494), **(C)** *Pseudomonas aeruginosa* (CCT 1476), and **(D)** *Escherichia coli* (CCT1457).

A

Results

			Mean (mV)	Area (%)	St Dev (mV)
**Zeta Potential (mV):**	-27,9	**Peak 1:**	-28,0	100,0	5,16
**Zeta Deviation (mV):**	4,95	**Peak 2:**	0,00	0,0	0,00
**Conductivity (mS/cm):**	0,280	**Peak 3:**	0,00	0,0	0,00
**Result quality**	Good				

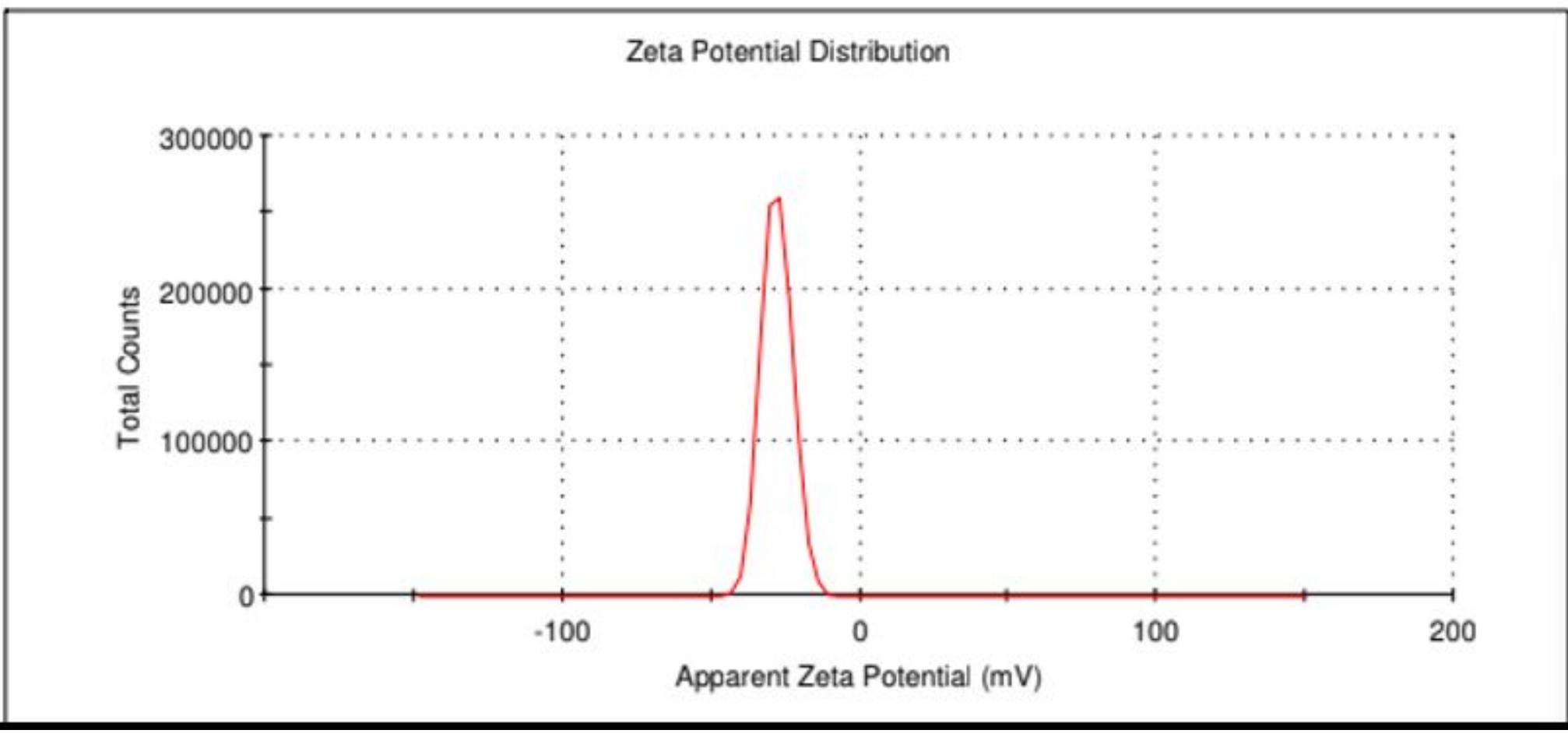

B

Results

			Mean (mV)	Area (%)	St Dev (mV)
**Zeta Potential (mV):**	-29,2	**Peak 1:**	-29,2	100,0	5,97
**Zeta Deviation (mV):**	5,97	**Peak 2:**	0,00	0,0	0,00
**Conductivity (mS/cm):**	0,258	**Peak 3:**	0,00	0,0	0,00
**Result quality**	Good				

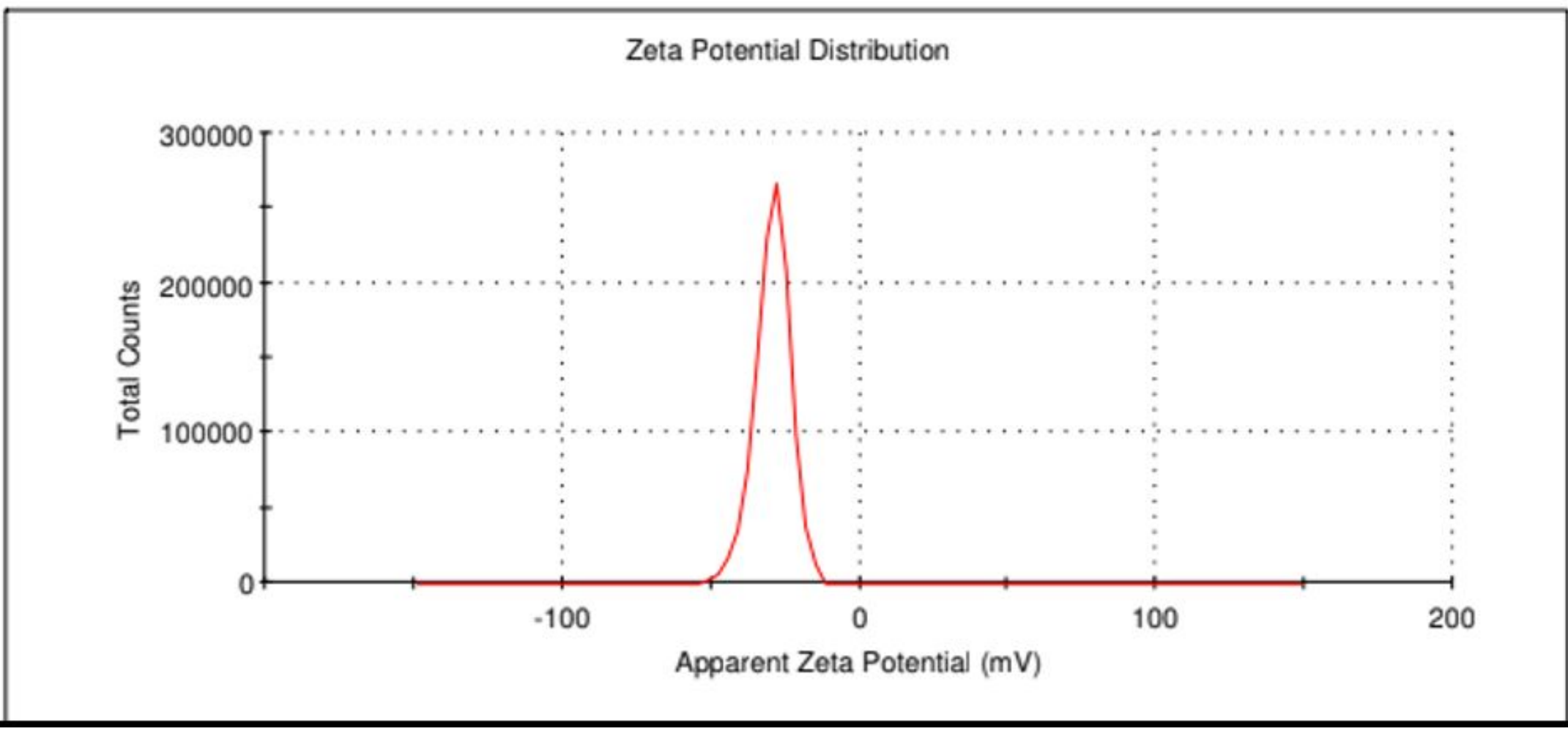

C

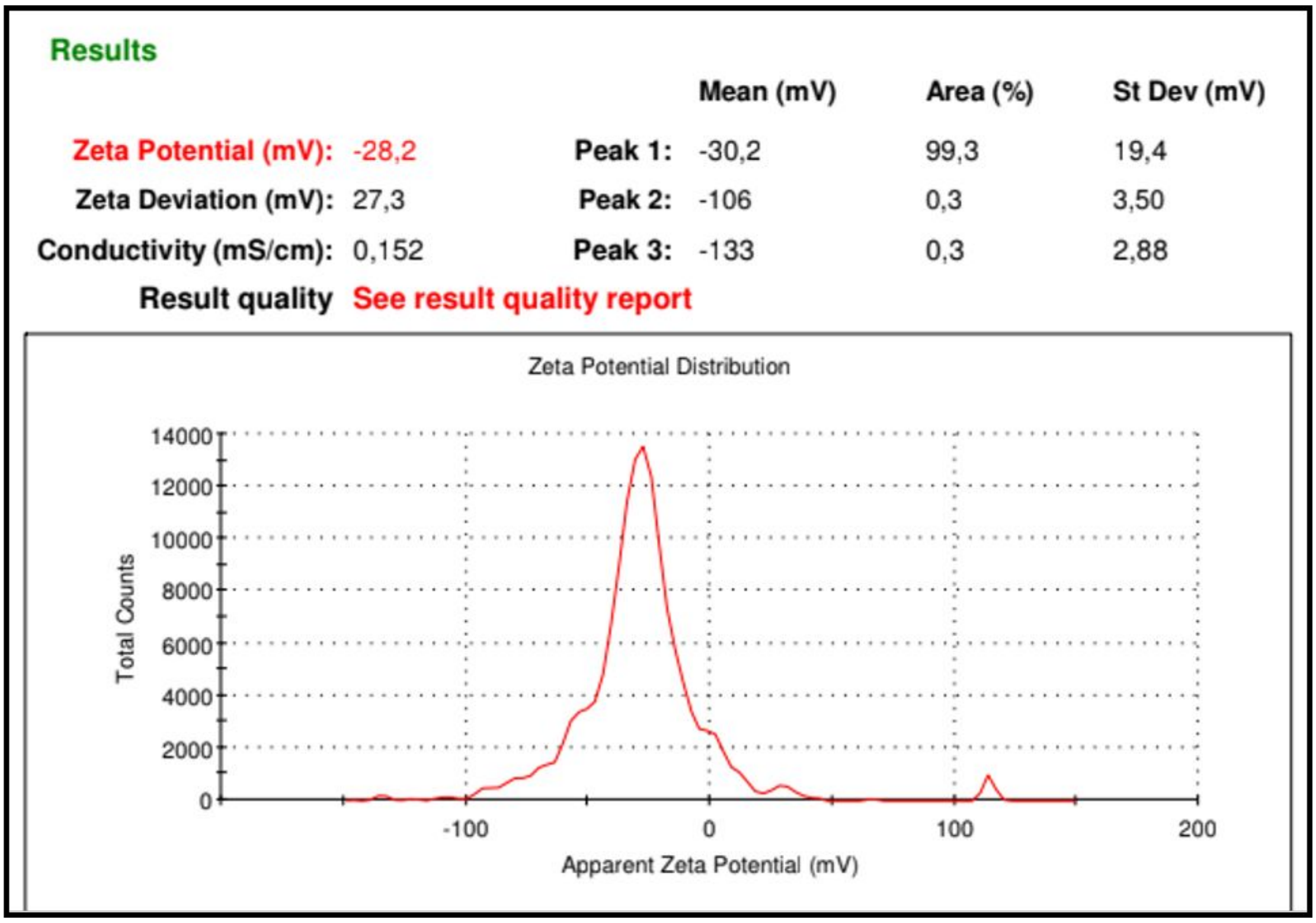

D

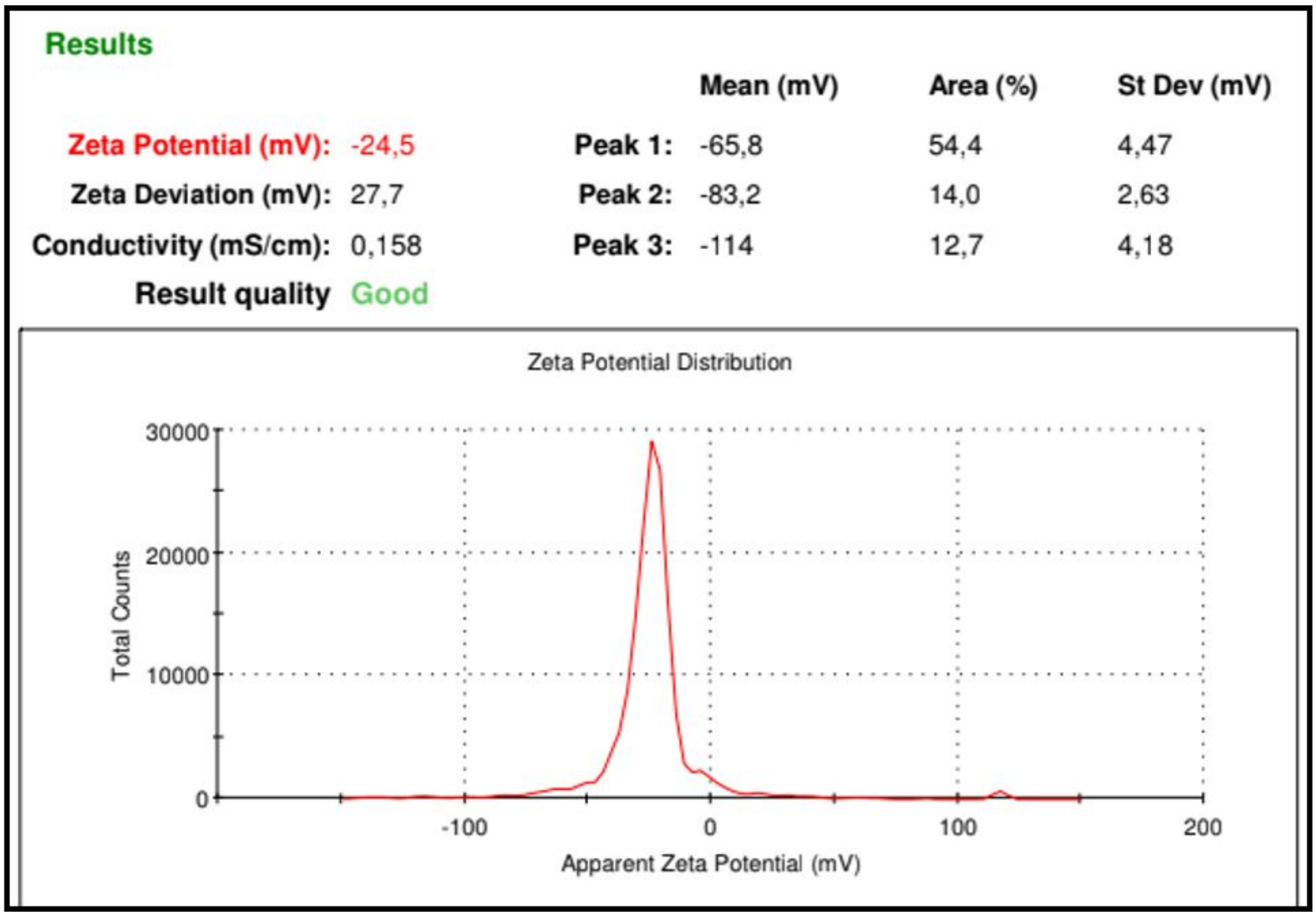

Fig. S2 - Zeta potential analyzed by DLS: (**A**) 1 day, (**B**) 30 days, (**C**) 60 days, and (**D**) 90 days.

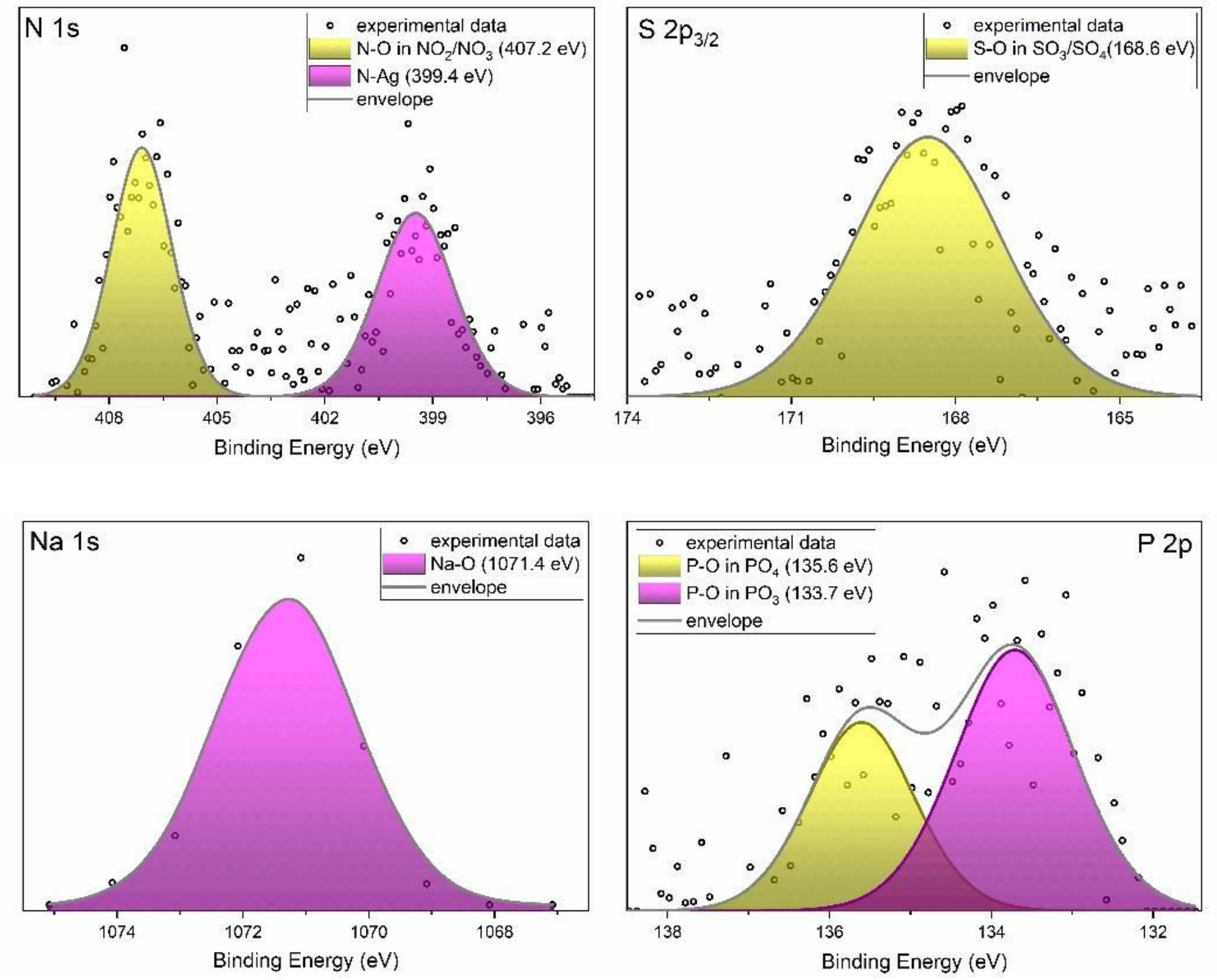

Fig. S3 - Typical examples of deconvoluted N 1s, S $2p_{3/2}$, Na 1s, and P 2p lines from the XPS spectra of AgNP@SA. Component peak positions and possible identifications are indicated in the legends.

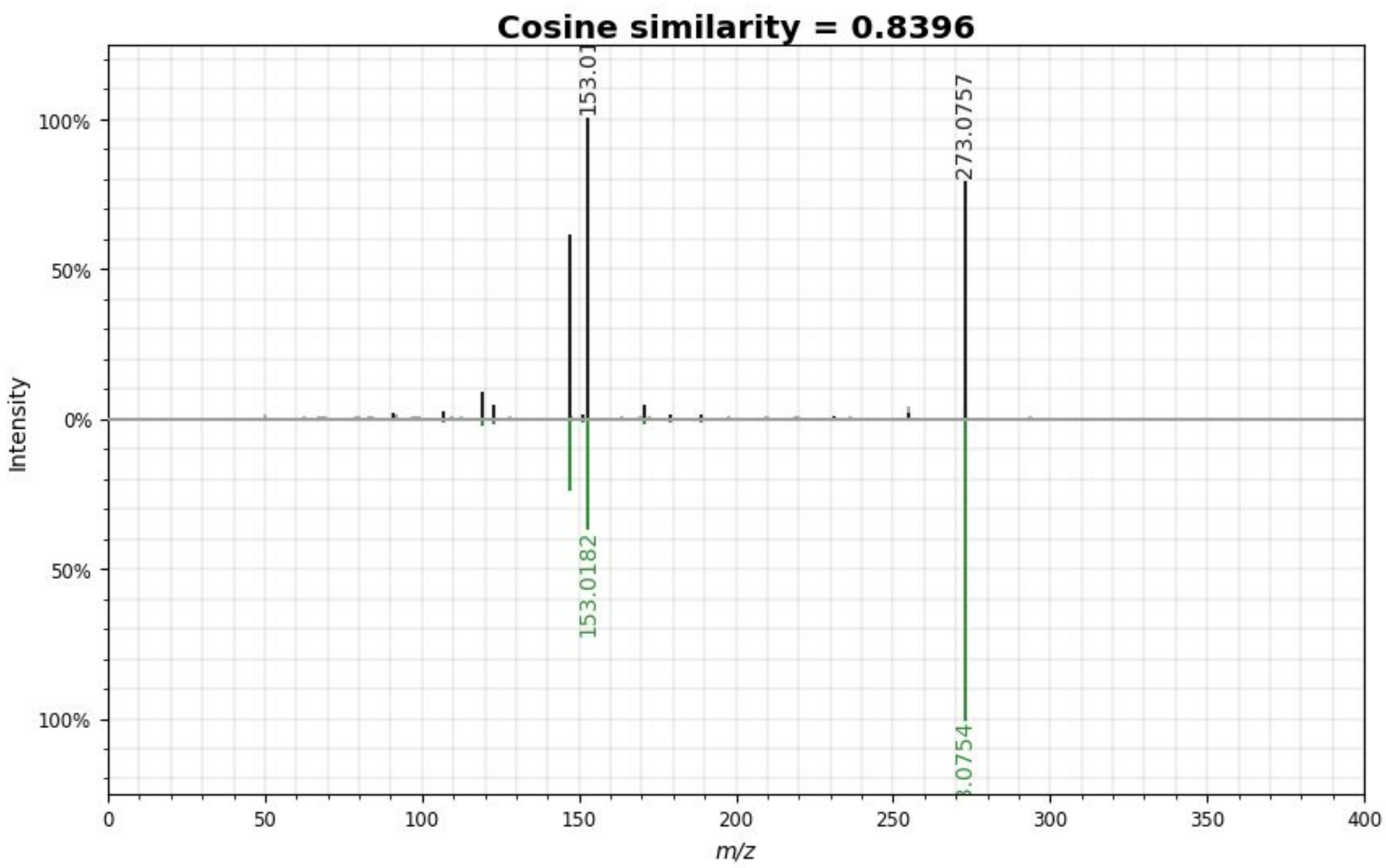

Fig. S4 - Mirror matching of *m/z* 273.0760 (**naringenin**) with the cosine similarity = 0.8396.

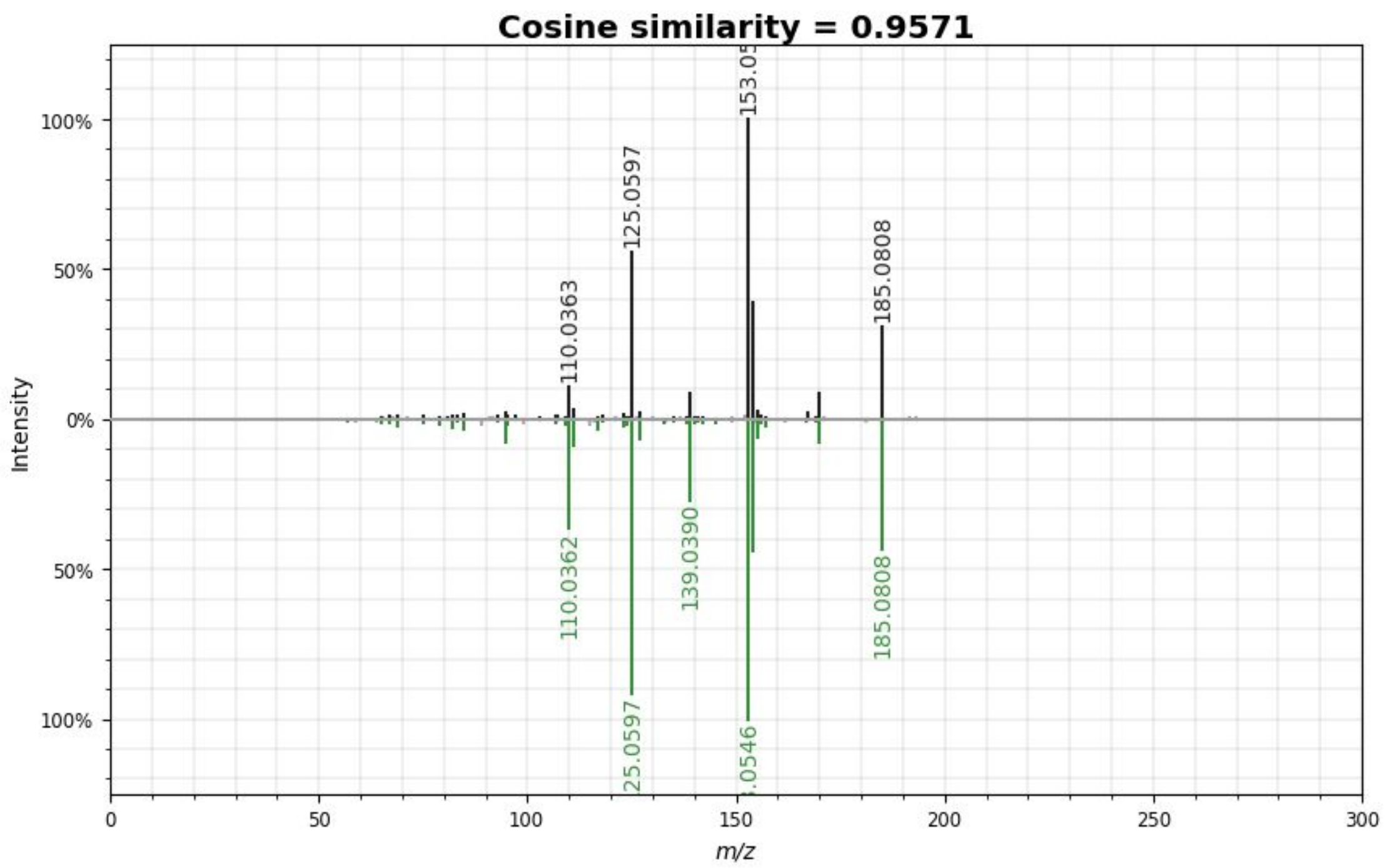

Fig. S5 - Mirror matching of *m/z* 185.0808 (**3,4,5-trimethoxyphenol**) with the cosine similarity = 0.9571.

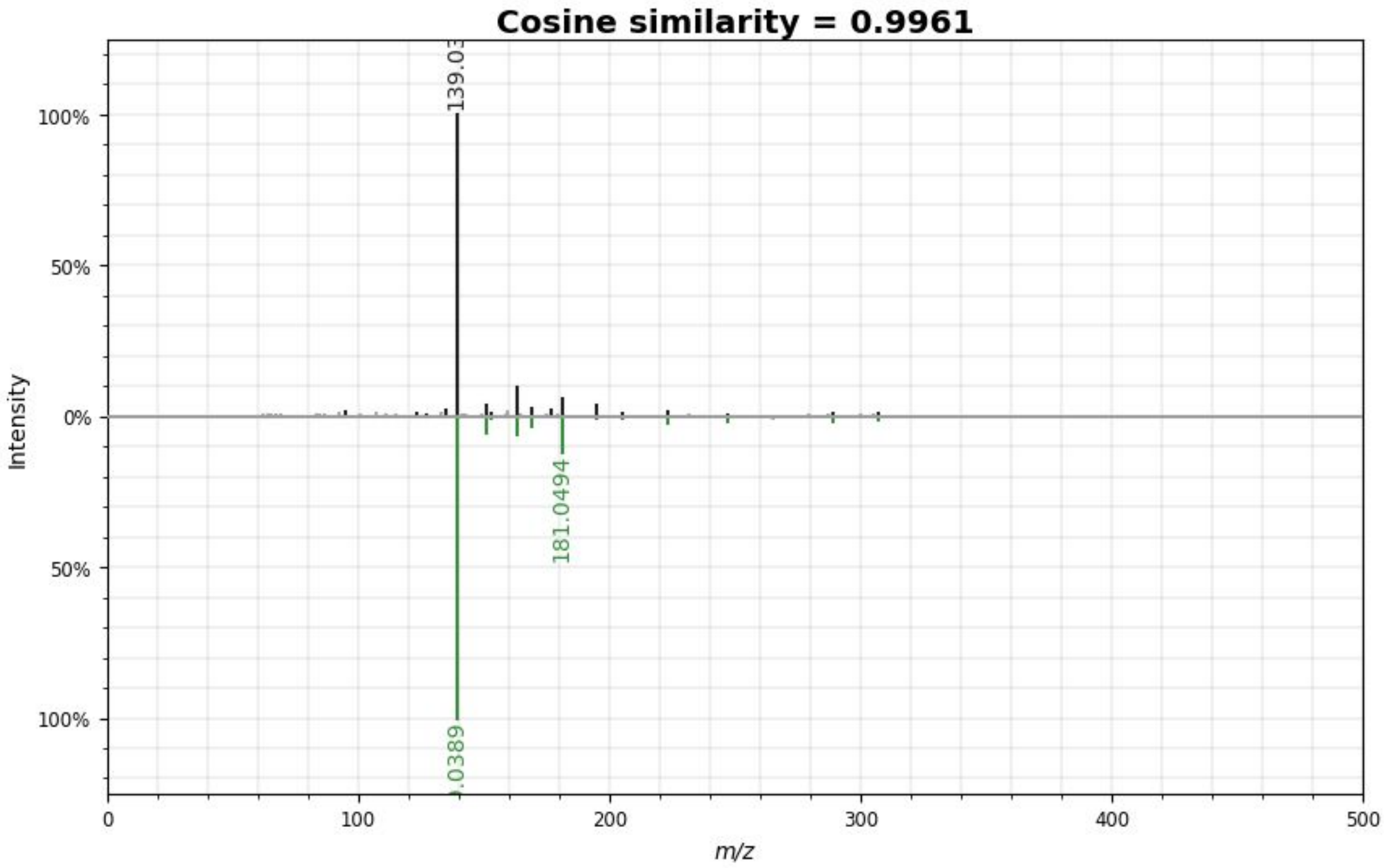

Fig. S6 - Mirror matching of *m/z* 307.0812 (**epigallocathechin**) with the cosine similarity = 0.9961.

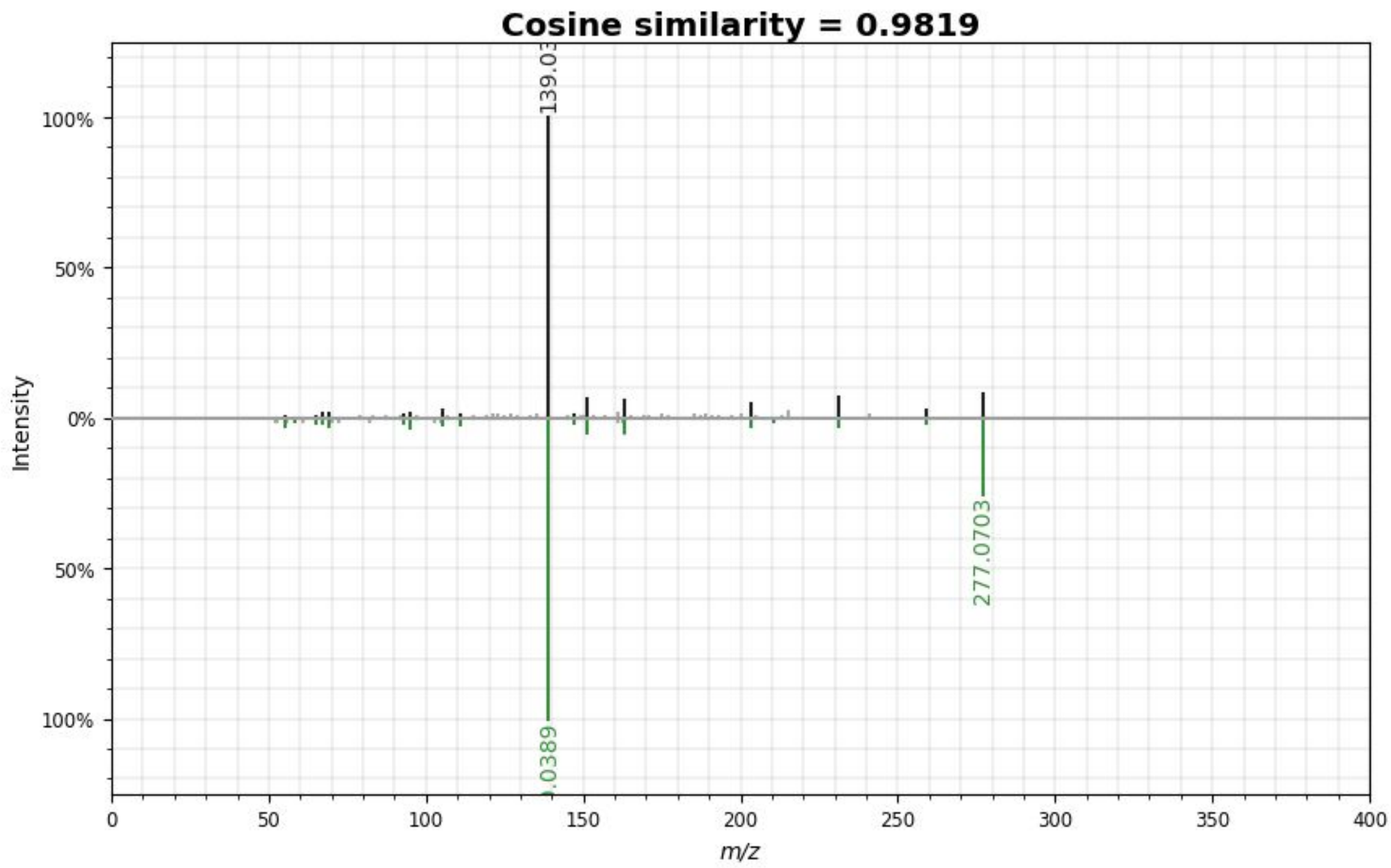

Fig. S7 - Mirror matching of *m/z* 277.0707 (**teadenol A**) with the cosine similarity = 0.9819.

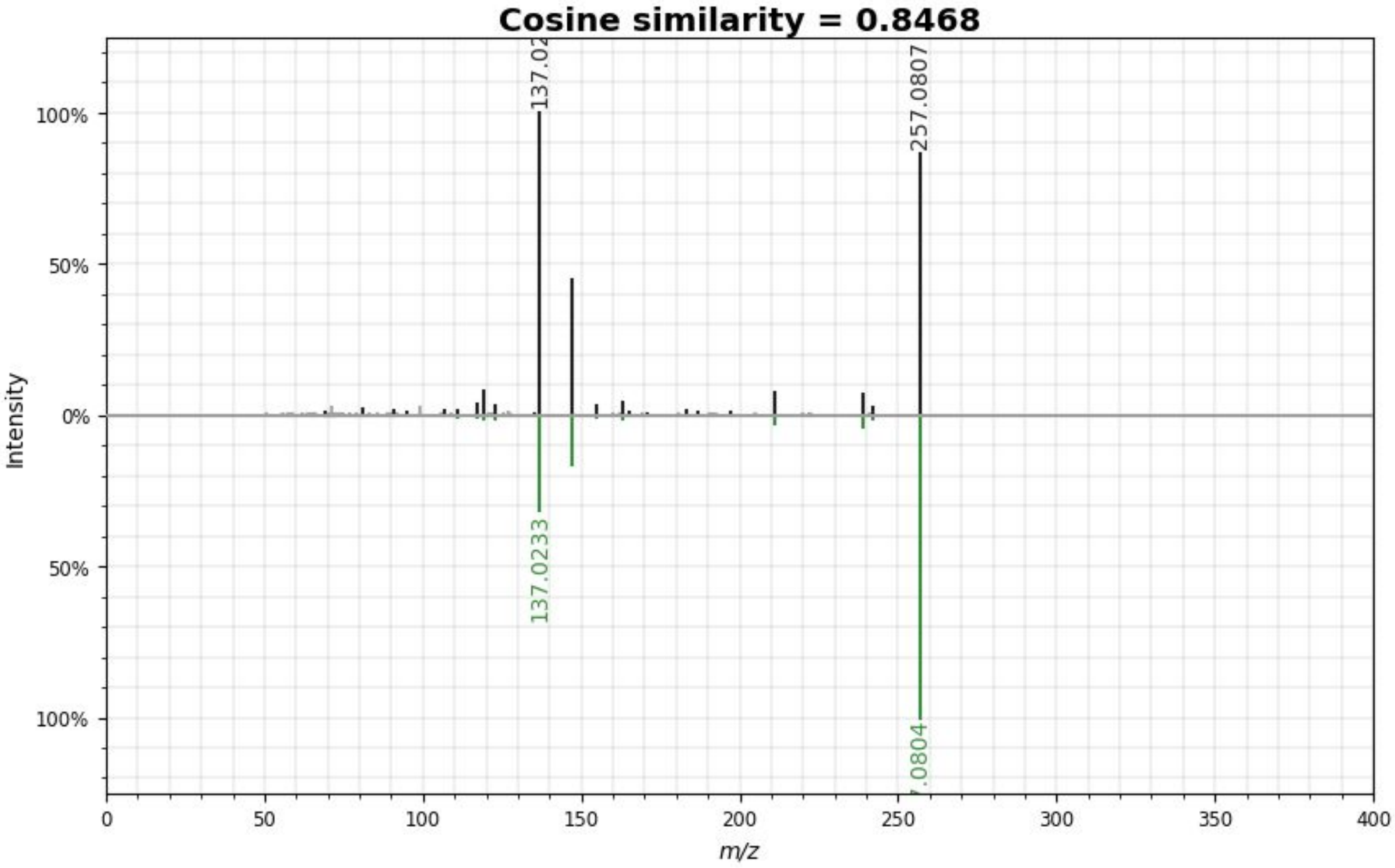

Fig. S8 - Mirror matching of *m/z* 257.0808 (**isoliquiritigenin**) with the cosine similarity = 0.9819.

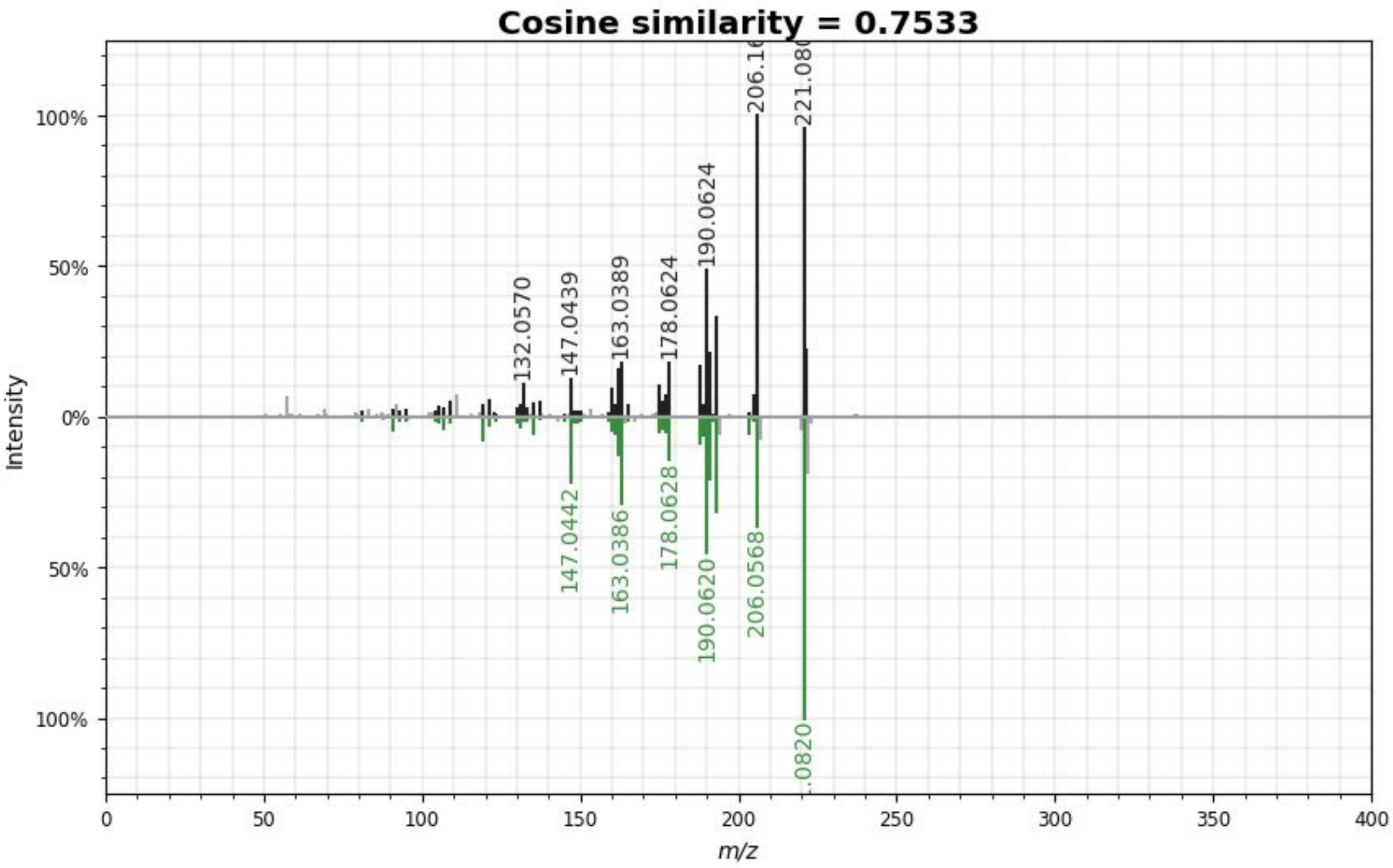

Fig. S9 - Mirror matching of *m/z* 239.0914 (**3,4,5 - trimethoxycinnamic acid**) with the cosine similarity = 0.7533.
